# Experiences of technology for increasing physical activity of older adults: a qualitative systematic review and meta-synthesis

**DOI:** 10.1186/s11556-025-00394-7

**Published:** 2025-12-05

**Authors:** Irem Huri Karabiyik, Aysegul Ilgaz

**Affiliations:** 1 Department of Public Health Nursing, Afyonkarahisar Sağlık Bilimleri Üniversitesi, Afyonkarahisar, Turkey; 2 Department of Public Health Nursing, Akdeniz University, Antalya, Turkey

**Keywords:** Older adults, Digital technology, Physical activity, Qualitative research

## Abstract

**Background:**

Technologies used to increase older adults’ physical activity encourage more daily movement. Innovative tools such as wearable devices, mobile apps, and virtual reality enhance exercise and motivation, enabling more targeted, personalized programs through health monitoring. While previous reviews explored the impact of technology on older adults’ physical activity, this study provides a synthesis of primary qualitative research focusing on older adults’ experiences with these technologies, offering an in-depth understanding of barriers, facilitators, perceptions of usability, motivations, and subjective effects. This meta-synthesis aimed to analyze, synthesize, and interpret the qualitative literature on older adults’ experiences with technology to increase physical activity.

**Methods:**

This meta-synthesis conducted a systematic literature search of PubMed, Scopus, CINAHL, and MEDLINE databases from inception to January 28, 2025. Data extraction and quality assessment were performed using the Joanna Briggs Institute Critical Appraisal Checklist for Qualitative Research (JBI-QARI). Data synthesis was carried out using the thematic synthesis process developed by Thomas and Harden (2008). The reliability and credibility of the findings were assessed using the ConQual approach. The protocol was registered in PROSPERO [CRD420251035985].

**Results:**

A total of 371 findings obtained from the 16 included studies were grouped into seven categories, resulting in two synthesized findings: (1) older adults’ pathways to meaningful technology use for physical activity and (2) structural and personal limitations in digital health engagement. The participants in all included studies were aged 60 years and older, and 61.4% were female. The technologies used to enhance physical activity included various approaches such as mHealth, telehealth, exergames, virtual reality (e.g., VR, VIMINT), wearable devices (e.g., FitBit, WAM), and digital exercise platforms (e.g., Connect 60+, Kikoeru, Alexa, ProAct65+).

**Conclusion:**

This study highlighted that older adults’ engagement with technology-based physical activity programs is supported by social support, accessibility, user-friendly designs, and personalized features, while it is hindered by low motivation, health or mobility issues, social isolation, environmental barriers, and technical difficulties. Tailoring interventions to individual needs, providing technical support, ensuring privacy, promoting social interaction, and developing safe, accessible, and culturally sensitive activities are essential. Future research should focus on creating inclusive technologies that enhance older adults’ physical activity.

**Supplementary Information:**

The online version contains supplementary material available at 10.1186/s11556-025-00394-7.

## Introduction

Older adults need to be more physically active for their independence, quality of life, and good aging [1]. According to the Centers for Disease Control and Prevention, “physical activity” can prevent or delay many health problems that occur with age [2]. Physical activity is defined as any bodily movement produced by skeletal muscles that requires the expenditure of energy [3]. Regular physical activity plays an effective role in the prevention and management of non-communicable diseases, such as cardiovascular diseases, cancer, stroke, hypertension, and diabetes, which are especially common among older adults. In addition, it contributes positively to individuals’ mental health and overall well-being [4–7]. In this regard, current guidelines recommend that individuals aged 65 years and older engage in ≥ 150 min of moderate-intensity or ≥ 75 min of vigorous-intensity aerobic physical activity per week, along with muscle-strengthening activities [3, 8].

Behavioral change therapies that successfully raise physical activity levels must be put into place to encourage healthy aging in older adults [9, 10]. However, traditional face-to-face health behavior interventions have limited impact due to time constraints, transportation difficulties requiring participants to visit facilities, adverse weather conditions, high costs, inadequate monitoring of daily behavior changes, and the need for professional expertise [11, 12]. Therefore, intervention approaches that are lower-cost, less human resource-intensive, and widely applicable are needed.

The advent of digital solutions presents a viable avenue for encouraging physical exercise among older persons, considering the drawbacks of conventional methods [13]. Technology is emerging as a popular tool for developing approaches that encourage older adults to participate in physical activity [14, 15]. Technological tools, such as smartphones, tablets, computers, wearable devices, and mobile applications, aim to increase individuals’ physical activity levels by providing digital solutions for behavioral change interventions [15, 16]. In addition, advanced technologies such as artificial intelligence (AI); blockchain; the Internet of Things (IoT); big data analytics; cloud computing; 5G networks; remote sensing; and geographic information systems (GIS) offer vast opportunities to increase the effectiveness, sustainability, and accessibility of such interventions by enabling comprehensive improvements in healthcare service delivery [17]. In this regard, technology-based interventions increase accessibility by overcoming barriers related to weather, time, and geography. They also provide a participatory physical activity experience for older adults by offering flexible and visual exercise resources, virtual community support, and more targeted, personalized programs [18–20]. Despite these benefits, participation in and adherence to technology-based physical activity interventions remain insufficient among older adults. Studies in Australia, the United States, and Switzerland have shown that around one-fifth of older adults use new technologies, such as online exercise classes, electronic wearables, and mobile tracking devices for physical activity [21–23]. Older adults who use these technologies are more likely to be younger, male, interested in technology, and engage in regular exercise [21].

The current systematic reviews have looked at how well technology increases physical activity [24–26], but it’s also critical to look into older persons’ subjective experiences, perceptions, and psychosocial reactions to these technologies. Additionally, examining how technology is incorporated into day-to-day activities offers important insights into the motives, difficulties, and meanings that influence physical activity participation [27, 28]. In the USA, a study found that older adults generally enjoyed using technology for exercise purposes, felt more accountable for their physical activity, experienced increased activity levels, and reported positive experiences. However, difficulties and negative experiences regarding the functionality and usability of the technology were also reported [29]. In Australia, a study exploring older adults’ perspectives on technology for promoting physical activity found that participants held positive perceptions and experiences regarding technology in terms of movement reminders, activity measurement, goal setting, monitoring long-term progress, data sharing, enjoyment, social interaction, and health benefits, while also reporting negative perceptions related to feeling too old to use technology, lack of training, and low confidence in its use [30].

Understanding how technology influences physical activity and experiences among older adults is vital to support healthy aging. Systematic reviews of quantitative studies evaluating the effect of technology on increasing physical activity in older adults are available [24–26, 31]. However, no study synthesizing qualitative studies on this topic has been found [32, 33]. Determining the technological needs, expectations, experiences and motivations of older persons can help create user-centered apps that improve the efficacy and sustainability of interventions. By integrating primary qualitative research findings from various contexts in a comprehensive manner, qualitative evidence synthesis now facilitates the creation of new conceptual models, the identification of research gaps, and the design of successful health interventions [34, 35]. This meta-synthesis aimed to analyze, synthesize, and interpret qualitative literature on older adults’ experiences with technology in increasing physical activity. The following research question was developed: “What barriers, facilitators, perceptions of usability, motivations, and subjective effects do older adults encounter when using technology to promote physical activity?”

## Methods

### Design

This meta-synthesis was conducted following the guidelines outlined in the Joanna Briggs Institute (JBI) Manual for Evidence Synthesis [36]. The qualitative systematic review protocol was registered with the International Prospective Register of Systematic Reviews (PROSPERO) database [registration protocol code: CRD420251035985]. The review process was guided by the Enhancing Transparency in Reporting the Synthesis of Qualitative Research (ENTREQ) framework [35]. The review results were standardized according to the Preferred Reporting Items for Systematic Reviews and Meta-Analyses (PRISMA) guidelines [37]. To ensure methodological rigor in this meta-synthesis process, all guidelines were strictly followed.

### Search strategy

The search strategy, focusing on experiences related to the use of technology for increasing physical activity levels among older adults, was developed through joint evaluation and consultation by the research team. Both Medical Subject Headings (MeSH) terms and free-text keywords were used, with the search terms reviewed and optimized through an iterative process. The search terms were organized into four main concepts: (1) older adult, (2) physical activity, (3) technology, (4) qualitative research. The search terms developed based on these concepts were combined using Boolean operators (AND, OR, NOT). A comprehensive systematic searches were conducted in four databases: PubMed, Scopus, CINAHL, and MEDLINE from inception to January 28, 2025, without any year restrictions. The detailed search strategy applied in each database is presented in Additional file 1. In addition, the reference lists of studies meeting the inclusion criteria were manually screened. None of the research assessed for inclusion in the reference list, however, fulfilled the requirements beyond those listed in the databases.

The SPIDER (Sample, Phenomenon of Interest, Design, Evaluation, Research type) tool, designed for qualitative and mixed methods research, was used in this meta-synthesis [38]. The SPIDER was chosen because it provides a more effective basis for qualitative and mixed methods research queries than PICO. This approach allowed the screening process to be more strongly linked to the qualitative literature, making the process more efficient and sensitive by reducing the time required to analyze the search results [38]. The inclusion criteria were structured according to the SPIDER tool: (1) older adults aged 60 years and older; (2) technologies used to increase physical activity (e.g. wearable devices, mobile apps, smart watches, online programs); (3) qualitative study designs including interviews, focus groups, observations, case studies, or diary studies; (4) older adults’ experiences, attitudes, motivations, barriers and facilitators of technology use; (5) qualitative or mixed methods study (extracting qualitative section only), and published only in English with full text available (Table 1).

**Table 1:** SPIDER tool for qualitative evidence synthesis

SPIDER	Justification
S - Sample	Older adults aged 60 years and older.
PI - Phenomenon of Interest	Technologies used to increase physical activity (e.g. wearable devices, mobile apps, smart watches, online programs).
D - Design	Qualitative study designs including interviews, focus groups, observations, case studies, or diary studies.
E - Evaluation	Older adults’ experiences, attitudes, motivations, barriers and facilitators of technology use.
R - Research type	Qualitative or mixed methods study (extracting qualitative section only).

The following criteria were excluded: (1) individuals under 60 years of age, (2) traditional methods of increasing physical activity (e.g. face-to-face intervention), (3) quantitative studies, conference abstracts, reviews, theses, commentaries, case reports, protocols, and grey literature (4) publications languages other than English, (5) publications without full-text availability or incomplete data, (6) duplicate publications.

### Study selection

References obtained from the comprehensive literature review were imported into EndNote X20, and duplicate records were removed. The remaining references were then transferred to Rayyan software, where a double-blind screening was conducted to select studies systematically and transparently. First, two researchers independently screened titles and abstracts based on predefined inclusion and exclusion criteria to exclude studies unrelated to the topic. Second, to minimize the risk of missing relevant studies, the full texts of the remaining articles were independently evaluated by the same researchers. Finally, studies that fully met the inclusion criteria were selected for meta-synthesis. Disagreements that may arise between the two researchers during this process were resolved through discussion until consensus was reached based on inclusion and exclusion criteria to eliminate any inconsistencies. Therefore, there was no need for a third independent referee, and the two researchers came together and reached a mutual agreement. In order to reach an agreement on which articles to include, it was necessary to re-examine the search method, explain the inclusion criteria in detail, and review possible sources. Moreover, Cohen’s kappa coefficient was calculated to assess the consistency between the two researchers. Cohen’s kappa coefficient was determined to be 0.838, indicating a strong level of agreement [39]. Collaboration and consensus building among the authors played an important role in ensuring the integrity and reliability of the study selection process.

### Data extraction

Data were independently extracted by two researchers using the JBI Qualitative Data Extraction Tool for Qualitative Research (JBI-QARI) [40]. Researchers recorded the data extraction processes separately in the same standardized data extraction tables created in the Microsoft Excel template. This data extraction table contains the following information: author/year, country, methodology, participant characteristics, technology interventions, aim of the study, key findings (themes and subthemes). A systematic harmonization approach was adopted to ensure a consistent presentation of information when the same data were reported in different formats across studies. Where possible, the extracted data points were converted into a consistent format for readers (e.g., means and standard deviations for age). For mixed-method studies, only qualitative findings were extracted. The methodology of all studies included in the meta-synthesis during the data extraction process was adequately explained, and no studies with unsupported findings were encountered (Table 2). To handle interpretive subtleties and settle any possible inference inconsistencies, a systematic reconciliation procedure was put in place. After completing their independent analyses, the two researchers met to compare the data row by row, marking any potential differences on the data extraction table. Each difference was addressed through a structured three-stage discussion process: (i) each researcher presented their rationale for their own extraction, (ii) the validity of different interpretations was discussed, taking into account the contextual features of the findings (e.g., the purpose of the study, participant characteristics, data collection and analysis method), (iii) a common interpretation was reached, and this final decision was recorded in the data extraction table. Throughout the entire extraction and comparison process, the two researchers reached full agreement in all cases, with no instances of unresolved disagreement.

**Table 2:** Quality assessment of the included studies (*n*=16)

Citations	Q1	Q2	Q3	Q4	Q5	Q6	Q7	Q8	Q9	Q10	Score^a^	Rating^b^
(Li et al. 2024) [29]	Y	Y	Y	Y	Y	N	N	Y	U	Y	7	Moderate
(Hosseini et al. 2024) [51]	Y	Y	Y	Y	Y	N	N	Y	Y	Y	8	Moderate
(Dunston et al. 2024) [47]	Y	Y	Y	Y	Y	N	Y	Y	Y	Y	9	High
(Akinrolie et al. 2024) [50]	Y	Y	Y	Y	Y	Y	N	Y	Y	Y	9	High
(Zytnick et al. 2023) [48]	Y	Y	Y	Y	Y	N	Y	Y	Y	Y	9	High
(Weselman et al. 2023) [53]	Y	Y	Y	Y	Y	N	Y	Y	Y	Y	9	High
(Stawarz et al. 2023) [55]	Y	Y	Y	Y	Y	N	Y	N	Y	Y	8	High
(Shinokawa et al. 2023) [56]	Y	Y	Y	Y	Y	N	N	Y	Y	Y	8	High
(Crane et al. 2023) [46]	Y	Y	Y	Y	Y	N	N	N	Y	Y	7	Moderate
(Jansons et al. 2022) [52]	Y	Y	Y	Y	Y	Y	Y	Y	Y	Y	10	High
(Simmich et al. 2021) [30]	Y	Y	Y	Y	Y	N	U	Y	Y	Y	8	Moderate
(O’Brien et al. 2021) [57]	Y	Y	Y	Y	Y	N	N	N	Y	Y	7	Moderate
(Wichmann et al. 2020) [58]	Y	Y	U	Y	Y	N	Y	N	Y	Y	7	Moderate
(Abouzahra and Ghasemaghaei 2020) [49]	Y	Y	Y	U	Y	N	N	Y	Y	Y	7	Moderate
(Maula et al. 2019) [54]	Y	Y	Y	Y	Y	N	N	N	Y	Y	7	Moderate
(Ehn et al. 2019) [59]	Y	Y	Y	Y	Y	Y	Y	Y	Y	Y	10	High

*Abbreviations:Y* yes, *U* unclear, *N* no, *a* JBI Critical Appraisal Tool score, *b* JBI ConQual score (Q2, Q3, Q4, Q6, Q7), *Q1* Is there congruity between the stated philosophical perspective and the research methodology?, *Q2* Is there congruity between the research methodology and the research question or objectives?, *Q3* Is there congruity between the research methodology and the methods used to collect data?, *Q4* Is there congruity between the research methodology and the representation and analysis of data?, *Q5* Is there congruity between the research methodology and the interpretation of results?, *Q6* Is there a statement locating the researcher culturally or theoretically?, *Q7* Is the influence of the researcher on the research, and vice- versa, addressed?, *Q8* Are participants, and their voices, adequately represented?, *Q9* Is the research ethical according to current criteria or, for recent studies, and is there evidence of ethical approval by an appropriate body?, *Q10* Do the conclusions drawn in the research report flow from the analysis, or interpretation, of the data?

### Data synthesis

This study synthesized data using the “thematic synthesis” approach developed by Thomas and Harden (2008) for the systematic review of primary qualitative research [41]. This thematic synthesis process, consisting of three basic steps, was carried out as follows: findings were coded line by line according to their meaning and content (step 1); descriptive themes were developed based on the similarities and differences in meaning among the codes obtained (step 2); analytical themes explaining barriers, facilitators, motivations, perceptions of usability, and subjective effects were created based on the descriptive themes (step 3). This cyclical process was repeated until the new themes became abstract enough to define or explain all of our initial descriptive themes. On the other hand, data synthesis was structured using the meta-aggregation approach recommended by the JBI Manual for Evidence Synthesis [36]. Two researchers independently identified thematic statements, participant quotes, and observations that directly supported the primary qualitative findings through a thorough review of the included studies. Through constant comparison and adjustment, the findings were grouped according to similarities in meaning to form categories. Synthesized findings were obtained by analyzing the relationships among the resulting categories. Differences of opinion among the researchers were resolved through discussion and consensus-building. To ensure rigor, each finding was classified by the researchers as “Unequivocal,” “Equivocal,” or “Unsupported” [42]. This synthesis only contained studies with results categorized as “Unequivocal” or “Equivocal.” No studies with “Unsupported” data were found. The detailed credibility assessment process for each finding obtained regarding synthesized findings and categories is presented in Additional file 2.

### Appraisal of methodological quality

**Fig. 1:**
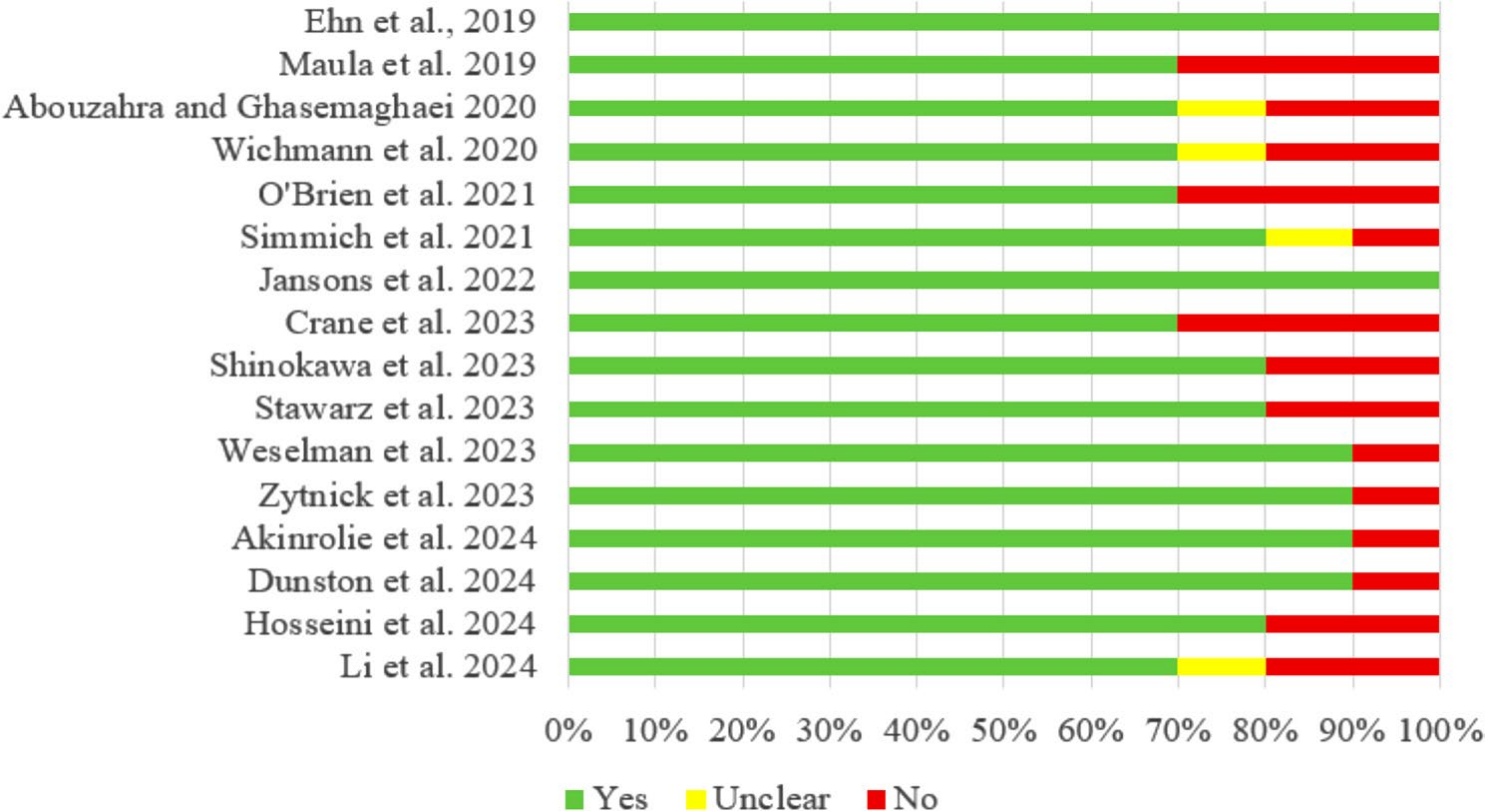
The distribution of risk of bias for each item on the JBI checklist

The quality of the included studies was independently assessed by two researchers using the JBI-QARI [43]. This tool, developed to assess the quality of qualitative studies, consists of 10 items, each rated “yes,” “unclear,” or “no.” Articles that received at least 60% “yes” responses from reviewers and were determined to be methodologically acceptable or of high quality were included in the meta-synthesis [44]. In this context, the risk of bias items for each included study are presented as percentages according to their fulfillment status (Fig. 1). The evaluations obtained after independent assessments conducted by the two researchers were compared, and in cases of disagreement, a consensus was reached through discussion. The quality appraisal results were used to determine the reliability level of the findings.

### Confidence of synthesised findings

The JBI-QARI assesses the methodological quality of included studies, while the ConQual approach assesses the confidence of synthesized findings through meta-aggregation [42]. In this meta-synthesis, the ConQual approach recommended by the JBI Manual for Evidence Synthesis was adopted to systematically assess the reliability and credibility of synthesized qualitative findings. According to this approach, the reliability of the studies included in the meta-synthesis is assessed using JBI-QARI. Five items in this checklist (Q2, Q3, Q4, Q6, and Q7) relate to the concept of reliability. Reliability was graded as (i) high (if 4–5 items were met), (ii) medium (if 2–3 items were met), and (iii) low (if 0–1 items were met). Credibility is classified as (i) unequivocal (clearly supported by data), (ii) equivocal (open to interpretation), and (iii) unsupported (lacking data support). If the synthesized findings consist solely of definitive conclusions, their credibility was considered high. Credibility was downgraded by one level for findings containing both clearly supported and interpretable data; by two levels for findings containing only interpretable data; by three levels for findings containing both interpretable and unsupported data; and by four levels for findings containing only unsupported data [34]. Ultimately, the overall ConQual scores were rated as high, moderate, low, or very low.

### Reflexivity

Reflexivity plays a crucial role, as researchers’ perspectives, experiences, positions, and assumptions can potentially influence the stages of the meta-synthesis process [45]. The first researcher is a female public health nurse with two years of professional experience and completed a master’s thesis employing a qualitative research design. The researcher’s studies focus on older adults’ access to healthcare services and participation in physical activity, and qualitative research has been published on these topics. The second researcher is a public health nurse specialist with 16 years of clinical and academic experience, whose doctoral dissertation focused on loneliness in older adults. The researcher’s studies covers a variety of topics, including older adult participation in physical activity, self-neglect, successful aging, aging technologies, and the provision of healthcare services for older immigrants, and qualitative studies have been published in these areas. In addition, both researchers have received training in qualitative research and hold certificates. The accumulated knowledge of both researchers provided an in-depth perspective in synthesizing the data. However, their prior areas of interest and assumptions naturally created potential bias in the analysis process. For this reason, both researchers adopted a continuous reflexive approach throughout the study. They clearly acknowledged their own biases and assumptions and carried out the analysis in a systematic and transparent manner. Moreover, the collaboration and discussions between the researchers strengthened both methodological and personal reflexivity, thereby helping to reduce the influence of subjectivity in the interpretation of the findings.

## Results

### Literature search

**Fig. 2:**
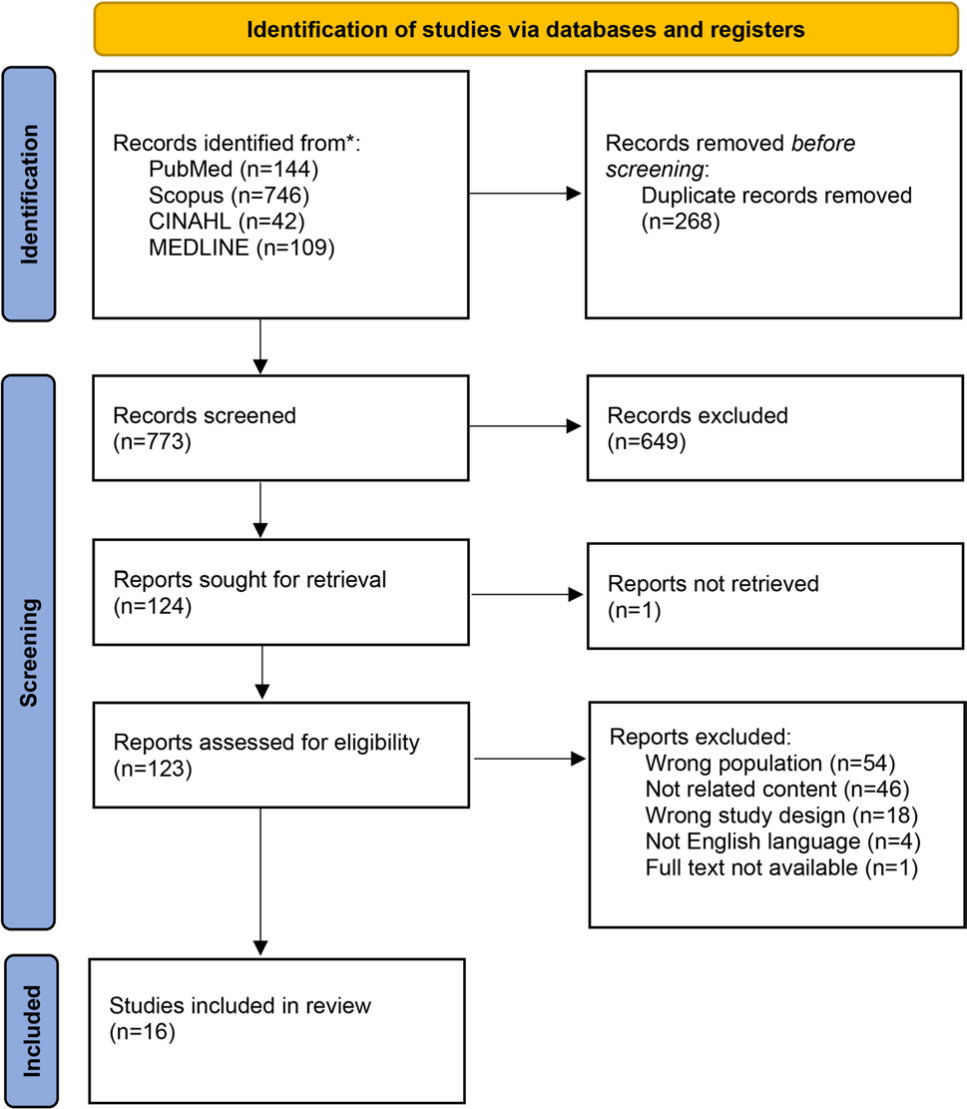
PRISMA flowchart

The initial search yielded 1041 studies. After removing duplicates, 773 unique studies remained. Title and abstract screening excluded 649 studies. Following full-text review of the remaining 122 studies, studies were excluded due to reasons such as inappropriate population, irrelevance to the topic, non-qualitative study design, and not being in the English language. Since the full text of one article could not be accessed, an e-mail was sent to the authors, but no response was received. Consequently, 16 studies were included in this meta-synthesis. The literature search process, which followed the PRISMA guidelines, was presented in Fig. 2.

### Quality appraisal

According to the JBI Critical Appraisal Tool score assessment for the 16 studies included in the meta-synthesis, the quality scores of the included studies ranged from 7 to 10. In addittion, ConQual score was calculated and eight studies were rated as high quality. The remaining studies were rated as moderate quality due to insufficient reporting of the researcher’s theoretical/cultural positionality or their interaction with the research process. Table 2 presents the quality appraisal results of the included studies.

### Study characteristics

Table 3 summarizes the characteristics of the 16 included studies. These studies were published between 2019 and 2024. The total sample size consisted of 282 participants, of whom were 61.4% were female (*n* = 173), and 38.6% were male (*n* = 109). Studies were conducted in the United States of America (USA) (*n* = 4) [29, 46–48], Canada (*n* = 3) [49–51], Australia (*n* = 3) [30, 52, 53], United Kingdom (*n* = 2) [54, 55], Japan (*n* = 1) [56], Ireland (*n* = 1) [57], Germany (*n* = 1) [58], and Sweden (*n* = 1) [59]. The studies comprised qualitative (*n* = 14) [29, 30, 46–55, 57, 59], and mixed-method (*n* = 2) [56, 58]. The predominant methodology employed was the descriptive qualitative method [29, 48, 50, 51, 56]. Data collection methods included semi-structured individual interviews (conducted face-to-face, by telephone, or via videoconference) [29, 30, 49–58] and focus groups [46–48, 59]. Data analysis methods included content analysis (*n* = 6) (29, 30, 50, 56, 58, 59), thematic analysis (*n* = 8) [47–49, 52, 53, 55, 57, 59], interpretive description (*n* = 1) [51], and framework analysis (*n* = 1) [54]. NVivo software was used in six studies during the data analysis process [29, 30, 48, 51, 52, 54]. Technology interventions used to increase physical activity among older adults included some methods such as mhealth, Virtual Reality (VR) Gaming, VIrtual Motivational INTerviewing (VIMINT), telehealth exercise program, Wearable Activity Monitor (WAM), Connect 60+ (a program that promoted exercise and social activities), Kikoeru app (allows older adults to share photos, calculate step counts and interact with other participants through voice recording), three-dimensional exergame, Amazon Echo Show 5 device (Alexa) (a home-based exercise program), wearable physical activity trackers, web-based programmes, wearable device (“FitBit”), ProAct65+, digital technology-based motivation support.

**Table 3:** Summary of the characteristics of the included studies (*n* = 16)

Author, year	Country	Methodology	Participant characteristics	Technology interventions	Aim of the study	Key findings (themes and subthemes)
(Li et al. 2024) [29]	USA	Qualitative study Descriptive Semi-structured interview (face-to-face) Content analysis (NVivo)	*n* = 23 60 years or older 18 female and 5 male	mHealth technology	To explore older adults’ acceptance, ability, and experiences of using three different types of electronic wearable devices for the purpose of self-monitoring and promoting physical activity over a 4–24-week period.	• Acceptance o Hedonic motivation • Capability o Effort expectancy o Habit and adherence o Facilitating conditions • Experience o Device learning o Performance expectancy
(Hosseini et al. 2024) [51]	Canada	Qualitative study Interpretive description Semi-structured interview (face-to-face) Interpretive description analysis (NVivo)	*n* = 15 aged 65 to 93 years 8 female and 7 male	Virtual Reality (VR) gaming	To explore how participants experience Virtual Reality (VR) exergames and the meaning they associate with their participation and to examine the factors that influence the experience of VR exergames and to investigate how these factors influence the overall experience.	• Enjoyment, excitement, and the novel environment • PA and motivation to exercise • Social connection and support • Individual preferences and challenges
(Dunston et al. 2024) [47]	USA	Qualitative study Semi-structured interview (face-to-face) Focus group Thematic analysis	*n* = 14 aged 65–79 years 12 female and 2 male	One-on-one telehealth exercise program	To identify barriers and facilitators to older cancer survivors’ (OCS) participation in telehealth-delivered exercise.	• Having adequate space to exercise o Facilitator 1a: Exercising in their own home o Barrier 1b: Limited space to exercise • Meeting OCS physical and psychosocial needs o Facilitator 2a: Individualization of the program o Facilitator 2b: Accountability o Facilitator 2c: Emotional and instrumental support • Older survivors learning throughout the exercise program o Facilitator 3a: Learning how to exercise with cancer o Barrier 3b: Learning curve with technology
(Akinrolie et al. 2024) [50]	Canada	Qualitative study Descriptive Semi-structured interview (face-to-face) Content analysis	*n* = 7 aged 65 years or older 5 females and 2 males	VIrtual Motivational INTerviewing (VIMINT)	To explore the experiences of older adults involved in virtual motivational interviewing (MI).	• Benefits and limitations of using technology o Positive communication platform experience o Technological limitations o Advantages of being in one’s environment o Convenient and flexibility o Virtual disadvantages o Positive virtual experience o Virtual limitation o Technological issue o Convenient factor o Safety concerns • Relationships between older adults and counsellors o Positive interaction with participant o Impact of virtual on rapport and relationship with participant o Positive characteristic of counsellors o Positive experience with counsellor o Undesirable characteristics of counsellor • MI skills and spirit o Virtual enhances MI skill o Easy to adhere to MI principle • Effects of virtual MI o Improved PA and self-awareness o Improved self-efficacy
(Zytnick et al. 2023) [48]	USA	Qualitative study Descriptive Discussion guide (face-to-face) Focus group Thematic analysis (NVivo)	*n* = 57 aged ≥ 60 years 44 female and 13 male	Wearable Activity Monitor (WAM)	To identify understand wearable activity monitor (WAM) use and its relationship with physical activity (PA) among older adults.	• Perceived Impact of WAM Use on PA among Ever WAM Users • Facilitators of Continued Use of WAM among Ever WAM Users • Reasons for Non-Adoption of WAM among Never WAM Users
(Weselman et al. 2023) [53]	Australia	Qualitative study Descriptive phenomenological approach Semi structured interviews (telephone) Thematic analysis	*n* = 13 age ≥ 65years 8 female and 5 male	Connect 60+ - a program that promoted exercise and social activities -	To explore the lived experience of older adults who participated in Connect 60 + delivered from a community hub that could be attended either in person or online.	• An enabling program design o Welcoming group facilitators o Variety of activities o Low cost o Community location o Online delivery • Developing new connections in the community o Social connections in the community o Sex mix o COVID-19 pandemic • Experiencing motivation to engage o Motivation to be fit and healthy o Understanding healthy aging
(Stawarz et al. 2023) [55]	United Kingdom	Qualitative study User-centered design (UCD) Semi-structured interview (face-to-face) Thematic analysis	*n* = 16 aged 69–89 years 10 female and 6 male	Exercise snacking and Tai Chi snacking movements	To explore how the novel exercise snacking approach, that is, incorporating short bouts of strength and balance activities into everyday routines, could be supported by technology within a home setting and what types of technologies would be acceptable for older adults who are prefrail.	• Attitudes Toward Exercise o Barriers to exercise o Exercise considered as important o Potential of exercise snacking • Exercising in the Home Environment o Impact of location o Safety o Cues to exercise • Opportunities and Challenges of Using Technology at Home o User expectations o Need to consider the context of use o Opportunities for exercise snacking technologies • Participants’ Use of Technology • Importance of Design Esthetics and Reliability • Challenges With Fitting the Device Into Everyday Life • Need for Personalization • Future Opportunities
(Shinokawa et al. 2023) [56]	Japan	Mixed-method study Qualitative Descriptive Design Semi-structured interview (face-to-face) Content analysis	*n* = 9 aged 65 and over 9 male	Kikoeru app (allows older adults to share photos, calculate step counts and interact with other participants through voice recording).	To verify the effectiveness of an application (app) in establishing social connectedness among unacquainted older men, as well as improving their physical health.	• Sharing Posts Deepens Understanding and Impressions o Development of greater respect for others o Listening to each other’s posts deepened their understanding of the members o Self-projecting onto other members’ posts and finding them relatable • Familiarity Advances the Relationship o Familiarity with the members, by way of knowing what they are doing, later led to deeper face-to-face conversations o Interaction through the app laid the foundation for face-to-face conversation o The desire to become acquainted with each other grew during interactions through the app o Sharing posts led to the desire to keep the group active o Feeling a sense of familiarity through online interaction, even without meeting • Being Interested in the Unfiltered Lives of the Members o Wanting to know the voice of the members, not just see posts that look good o Thinking about the members after seeing the posts and number of steps o Wanting to share things that look good, not just daily life activities o Feeling pressured to make a proper post o Wanting to positively impact other members through the app o Feeling glad about other members commenting on the posts o Feeling lonely when there’s no reaction to their post • Feeling Motivated to Stay Active o Wanting to challenge each other with the number of steps walked and make changes in their own life o Wanting to get a reaction to their own daily activities and being able to sympathize with members • Wanting More Than the Level of Interaction Through the App Alone o The relationship cannot be developed by online interaction alone
(Crane et al. 2023) [46]	USA	Qualitative study Semi-structured interview (face-to-face) Focus group Inductive and deductive analysis	*n* = 14 Aged 63–96 years 10 female and 4 male	Three-dimensional Exergame	To examine older adults’ motivators and barriers to joining and completing a three-dimensional exergame study.	• Motivators to join o Generativity o Peer referrals o Self-improvement o Curiosity • Motivators for retention o Accomplishment o Immersion o Exercise o Structured Schedule o Adaptive difficulty • Barriers to participation o Frustration o Fatigue/pain o Camera tracking issues o Gender-based trends o Engagement with study team o Commitment
(Jansons et al. 2022) [52]	Australia	Qualitative study Semi-structured interview (videoconferencing) Thematic analysis (NVivo)	*n* = 15 aged 60–89 years 9 female and 6 male	Amazon Echo Show 5 device (Alexa) 12 week	To evaluate the enablers and barriers for older adults participating in a home-based exercise program delivered and monitored by VIPAs.	• Enablers of interacting with the Alexa to participate in the exercise intervention o Voice interaction o Alexa device design o Ease of use and enjoyability o Motivation to participate in the exercise program o Social engagement with Alexa o Screen and voice compatible reminders • Barriers to interacting with the Alexa to participate in the exercise intervention o Technical issues o Preferences for other existing telehealth modes of exercise delivery o Lack of real-time objective feedback in an asynchronous digital health approach o Inadequate attention to patient goals in an asynchronous digital health approach o Privacy concerns
(Simmich et al. 2021) [30]	Australia	Qualitative study Exploratory approach Semi-structured interview (face-to-face or telephone) Content analysis (NVivo)	*n* = 19 Age (years), mean (SD): 70 (6) 11 female and 8 male	Wearable physical activity trackers	To explore perceptions about wearable physical activity trackers; perceptions about using technology to share physical activity information with clinicians; barriers and motivators to playing games, including AVGs for rehabilitation.	• Participant overview and technology use o Use of technology o Challenges of technology o Wearable activity trackers o Wanting to quantify o Helping to see long-term improvement o Allowing for clearer goal-setting o Reminders to be active o Concerns around social features • Sharing data with clinicians via technology o Perceived benefits of sharing data with clinicians o Concerns about sharing data with clinicians o Use of games • Socially-focused barriers and motivators o Fear of failure and embarrassment o Having fun with other people, especially family members o Not having anyone to play with o Competition is a double-edged sword • Self-focused barriers and motivators o A fun way to pass the time o Games being too difficult or risky o Not having the required equipment o Games as a form of beneficial exercise, or not o Games as a form of mental stimulation
(O’Brien et al. 2021) [57]	Ireland	Qualitative study Semi-structured interview (telephone) Thematic analysis	*n* = 11 Aged 60 years and over 11 female	Community-led walking programme “Step Up to Your Health”	To investigate the experiences and attitudes of older adults following a 6-week community-led walking programme utilising activity trackers.	• Programme as a Source of Motivation o motivator o more aware of activity levels o motivated by numbers o felt useful o changed behaviours o keeps active o loves walking/being outside anyway o inner motivation • User Experience of the Technology o required assistance with tracker set-up o no previous experience of this technology o belief technology gives you power o limits of tracker model used o experiences technical issues o easy of use of tracker o interesting/positive features of tracker o learning from the tracker o purchased or intends to purchase own tracker o curious of steps without tracker o continued usual walking routine • Views on Social Dimension of Programme o personal challenge/didn’t compare steps with others o element of competition o sharing experience with peers o desire for greater social dimension o disappointment at limited social dimension o not interested in social dimension o positive comparison with others o feeling embarrassed when comparing steps
(Wichmann et al. 2020) [58]	Germany	Mixed-method Semi-structured interview Content analysis	*n* = 25 Aged 65 years and older 15 female and 10 male	Web-based programmes (subprojects PROMOTE and RTC)	To evaluate the acceptance of a web-based PA program and identify requirements for future interventions.	• Requirements on interpersonal level • Requirements on intrapersonal or sociocultural level o Other participants and social Exchange o Trainers/Exercise instructors • Requirement on the content level of the proposed program (proposed program performed) o Exercises, instructions and goal setting o Group meetings o Specific eHealth intervention components • Requirements on spatial level o Accessibility/Reachability o Location • Requirements at the digital level • Requirements at the organizational level o Sequence and duration o Scheduling o Local stakeholders
(Abouzahra and Ghasemaghaei 2020) [49]	Canada	Qualitative study Semi-structured interview Thematic analysis	*n* = 26 age 65 and up 24 female and 20 male	Wearable device (“FitBit”)	To examine the factors that influence seniors’ use of wearable devices and the effect of these devices on seniors’ behavior.	• Technology Characteristics o Complexity o Alarms • Value o Activity monitoring o Motivation • Self-Efficacy and attitude towards technology o Self-Efficacy o Attitude towards technology • Social Influence o Family influence o Friends’ influence o Weak ties influence
(Maula et al. 2019) [54]	United Kingdom	Qualitative study Semi-structured interview (face-to-face) Framework analysis (NVivo)	*n* = 15 (FaME) *n* = 15 (OTAGO) 65 years and older FaME: 10 female and 5 male OTAGO: 12 female and 3 male	ProAct65 + trial: group (FaME) and home based (OTAGO) exercises	To provide a better understanding of physical activity maintenance behaviors in older people.	• Physical o Physical benefits o Deterioration in physical health o Health beliefs o Witnessing deterioration in others • Social • Social interaction • Motivating factors • Positive feedback from others • Habit • Carer role • Psychological o Measurable activity o Self-efficacy o Lack of time o Mental health • Environmental o Convenience o Follow on activity o Technology o Financial o Accessible transport o Weather o Safety
(Ehn, Johansson, and Revenäs 2019) [59]	Sweden	Qualitative study Explorative approach Semi-structured interview Focus group Content analysis Thematic analysis	*n* = 7 65 years and older 3 female and 4 male	Digital technology-based motivation support	To investigate older adults’ perceptions about the necessary contributions and qualities of digital technology-based motivational support for their physical activity.	• A help for the user in daily life o Surmountable o Customizable o Helpful facilitator • Strengthening motivation for PA, also among inactive persons o Conscious-raising o Making physical activity enjoyable o Useful for organizations reaching inactive persons

### Qualitative meta-synthesis

**Fig. 3:**
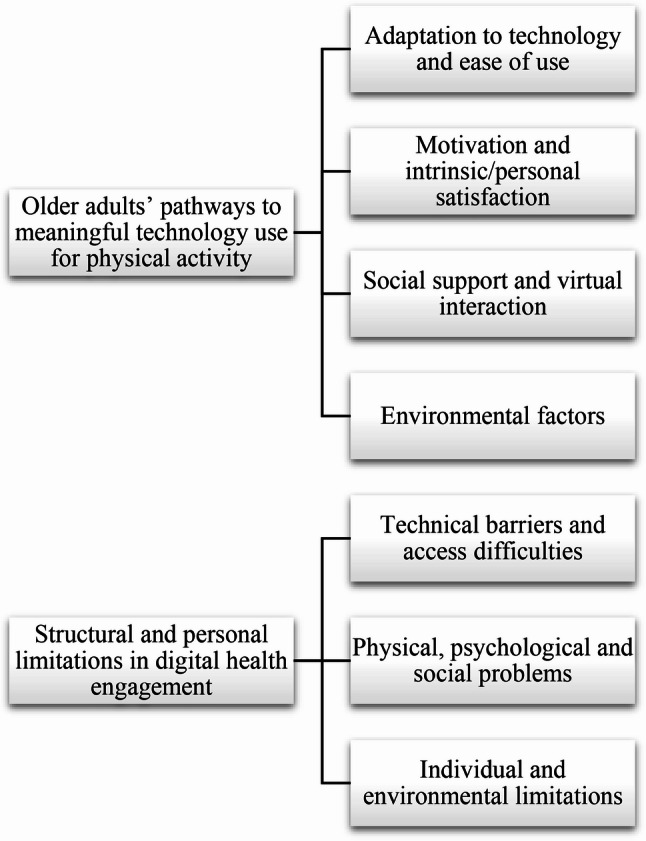
Meta-synthesis summary diagram

A total of 371 illustrative quotations were extracted from the 16 studies included in this meta-synthesis (Additional file 2). These quotations were converted into a total of 2 synthesized findings and 7 categories. The map of the synthesized findings and categories is presented in Fig. 3. The detailed distribution of the 16 included studies according to the synthesized findings and categories is presented in Table 4.

**Table 4:** The distribution of included studies according to synthesized findings and categories (*n* = 16)

Synthesised Findings	Categories	1	2	3	4	5	6	7	8	9	10	11	12	13	14	15	16
Older adults’ pathways to meaningful technology use for physical activity	Adaptation to technology and ease of use	✓	✓	✓	✓	✓	✓	✓		✓	✓	✓	✓	✓	✓		
	Motivation and intrinsic/personal satisfaction	✓	✓	✓	✓	✓	✓		✓	✓	✓	✓	✓	✓	✓	✓	✓
	Social support and virtual interaction			✓	✓	✓	✓		✓	✓	✓	✓	✓	✓		✓	✓
	Environmental factors			✓	✓		✓	✓					✓	✓		✓	✓
Structural and personal limitations in digital health engagement	Technical barriers and access difficulties	✓	✓	✓		✓		✓			✓	✓	✓	✓	✓		✓
	Physical, psychological and social problems	✓	✓		✓		✓	✓		✓	✓	✓	✓	✓		✓	✓
	Individual and environmental limitations		✓	✓	✓	✓	✓	✓	✓		✓	✓	✓	✓	✓	✓	✓

*Abbreviations:1* (Li et al. 2024) [29], *2* (Hosseini et al. 2024) [51], *3* (Dunston et al. 2024)[47], *4* (Akinrolie et al. 2024) [50], *5* (Zytnick et al. 2023) [48], *6* (Weselman et al. 2023) [53], *7* (Stawarz et al. 2023) [55], *8* (Shinokawa et al. 2023) [56], *9* (Crane et al. 2023) [46], *10* (Jansons et al. 2022) [52], *11* (Simmich et al. 2021) [30], *12* (O'Brien et al. 2021) [57], *13* (Wichmann et al. 2020) [58], *14* (Abouzahra and Ghasemaghaei 2020) [49], *15* (Maula et al. 2019) [54], *16* (Ehn et al. 2019) [59]

### Synthesised findings 1: older adults’ pathways to meaningful technology use for physical activity

#### Adaptation to technology and ease of use

Participants’ adaptation to technology emerged as a key facilitator of their participation in digital physical activity programs. Many participants reported that customizing wearable activity-tracking devices to their goals, needs, and schedules increased their level of compliance with the technology [29, 50, 51]. Some participants stated that they adapted to technology more easily because digital platforms provided convenience and flexibility [47, 50]. Additionally, several participants emphasized that the visual/auditory elements found on virtual platforms, such as vibrant colors, immersive environments, high-speed dynamics, and realistic design elements, enriched their technology experiences and made them more adaptable [51].

*“I think the good parts of it are*,* like I said*,* it’s more flexible*,* both for myself and for the participants [older adults] So*,* I can take the meeting anywhere I am*,* they can take the meeting anywhere they are…”(Akinrolie et al.*,* 2024; P1)* [50].

*“I feel like I’m facing real life*,* but the design is not quiet. I want to be immersed more in the game.” (Hosseini et al.*,* 2024; P9)* [51].

Participants reported that they had no problems using digital platforms, that they were very easy to use, and that these platforms were generally familiar, user friendly, and easily accessible [29, 51, 57]. A few participants emphasized that they could easily integrate such technologies into their daily lives, regardless of their technological proficiency levels, according to their physical capacity and living conditions [49]. They stated that they could easily track data on various daily activities, such as step count, heart rate, sleep patterns, swimming, and cycling [29, 48]. In addition, some participants underlined that features of digital assistants (Alexa, Buddy Link, Polar M600, etc.) such as their stable structure, small size, easy rechargeability, speech-based functionality, voice control, and user-specific content selection positively affected their technology experiences [29, 52].

*“The functions on the smartwatch were very clear… Charging was no problem. And then just swiping it and finding the information was very easy.” (Li et al.*,* 2024; P7)* [29].

*“It’s easy to use. There is a lot of exercises. You can skip what you don’t want. You do what you want and what you need. Those are all pluses.” (Jansons et al.*,* 2022; P1)* [52].

#### Motivation and intrinsic/personal satisfaction

Technology-based interventions aimed at increasing participants’ physical activity levels have been found to be motivating and to enhance their intrinsic/personal satisfaction levels. Most participants reported that wearable activity devices motivated them to be less sedentary in achieving their daily physical activity goals [48]. In addition, some participants explained that receiving daily rewards through technological tools for achieving these goals increased their self-awareness and enhanced their sense of intrinsic satisfaction [46, 48]. Several participants emphasized that technological tools with conversational functions, providing reminder and real-time feedback, increased their motivation to engage in physical activity. Some participants reported that based on their performance in the virtual game, they developed a more sustainable motivation toward technology-based interventions due to the effects of satisfying experiences, such as a sense of achievement, a desire to surpass themselves, and the development of their skills [46, 47].

*“When I look at my– I have done 3*,*649. I have 351 more steps to do before I get to my goal*,* and I’m going to do it.” (Li et al.*,* 2024; P8)* [29].

*“I’m walking more*,* because I know at five miles fireworks go off [on my WAM and…] I realize internally I love it.” (Zytnick et al.*,* 2023; P female)* [48].

*“I found it exciting!< LAUGHS >And I always wanted to do better*,* beat my score*,* or try to get to that next level. So it kept me coming.” (Crane et al.*,* 2023)* [46].

#### Social support and virtual interaction

Participants noted that telehealth-based digital exercise programs promoted positive group dynamics and that virtual interactions through these programs contributed to strengthening their social bonds [47]. Most participants highlighted that virtual interactions with other individuals were effective in increasing their physical activity levels by activating mutual support mechanisms [53, 54]. Some participants mentioned that when they used technological devices with a partner, they were more likely to maintain their physical activity levels by encouraging each other [58]. Participants who were less physically active also reported that chat-based technology interventions increased social support by providing an accessible communication environment. Many participants emphasized that the emotional and instrumental support provided by counselors, family members, and friends strengthened their adherence to digital programs [29, 48, 49, 51, 57]. In addition, sharing technology experiences increased participants’ self-efficacy and performance, and when combined with technical support and volunteer assistance, reinforced the potential of social support and information sharing in maintaining physical activity [51, 56].

*“Sometimes they can be really fun. When we have the family over […]*,* we have a Wii game […] You can have a lot of fun doing things like that. And that’s all movement and exercise too.” (Simmich et al.*,* 2021; P14)* [30].

*“The number of steps participant H takes daily is amazing. How does do it?” (Shinokowa et al.*,* 2023; PB)* [56].

#### Environmental factors

Some participants reported that being able to do exercises in their own home environment was a facilitating factor in terms of both sustainability and comfort in participating in physical activity [47, 58]. In addition, factors such as adequate space, the comfort of exercising in familiar surroundings, and the protection of individual privacy were among the factors that increased participation in physical activity. Furthermore, most participants underlined that they were more willing to continue their digital physical activity practices when environmental conditions were favorable [54].

*“Yeah*,* for me the fact that [it] is offered [by] distance*,* telemedicine*,* is the only reason I can do it ‘cause I can’t make the 6-hour round trip drive to go in-person.” (Dunston et al.*,* 2024; P69)* [47].

*“As long as it is sort of day time and you’re not travelling too far.” (Maula et al.*,* 2019; ID6)* [54].

### Synthesised findings 2: structural and personal limitations in digital health engagement

#### Technical barriers and access difficulties

Participants expressed that functional difficulties, such as complex interfaces, difficult device setup, complicated menu navigation, and unclear control buttons, were the main factors that negatively impacted their technology experience. In addition, technical obstacles such as voice recognition errors, devices not charging, camera tracking problems, connection drops, poor Internet connection, inaccurate measurement results, devices not synchronizing, and automatically deleting the day’s data at a certain time every day have negatively affected the user experience [29, 46–49, 52, 57]. Some participants reported needing support with technical and access issues, such as connecting to video calls, remaining in the camera’s field of view during exercise, and connecting with additional devices such as hearing aids [46, 52]. In addition, privacy and security concerns are technical barriers among participants. Several participants highlighted that fixed systems that cannot be customized to their personal needs exacerbate these concerns and negatively affect their participation in technological interventions. Therefore, they recommended that more in-depth training sessions should be organized that focus not only on device usage but also on technical issues, such as system security, data privacy, and personal information protection. Thus, it was highlighted that technical capabilities could be improved and concerns regarding privacy and security could be reduced [29, 46].

*“The voice interaction is very problematic. It does not understand a clearly enunciated yes or no*,* and I think there’s a reason for that. But you’ll see lots and lots of examples where it just refuses to understand a simple yes or no*,* or any other command*,* and given that it’s voice-driven that’s a critical failure.” (Jansons et al.*,* 2022; P6)* [52].

*“It took a while. I had to figure out how exactly to set up my ipad. Then move it when I went from floor to standing and that*,* but we’ve got it figured out now. But*,* it took a while.” (Dunston et al.*,* 2024; F*,* 72)* [47].

*“It did concern me*,* because you don’t want to think that you’re being listened to all the time and watched all the time*,* however it was alright once I found where to turn off the camera.” (Jansons et al.*,* 2022; P7)* [52].

#### Physical, psychological and social problems

Most participants reported shoulder, hip, knee, or back pain, along with fatigue, after their physical activity technology experiences [46, 54]. Some participants expressed that technological tools helped them gain self-awareness of physical inactivity that they had not previously noticed. Many participants emphasized concerns that digital physical activity interventions could be potentially hazardous due to requiring physically demanding actions [30, 51].

*“I was annoyed with myself that my left hand was not as accurate as my right hand. I’ve always been right-handed. I don’t use my left hand very much at all.” (Hosseini et al. 2024; P8)* [51].

*“When the grandkids were here they were doing some exercises…some exercise things on the…on the TV and I thought*,* ‘Oh*,* don’t think I could be doing that’. […] I have to be very careful what I’m doing because of my osteoporosis. Don’t want to start breaking bones otherwise I’m really badly off.” (Simmich et al. 2021; P13)* [30].

Many participants reported that psychological factors, such as depression, mental breakdown, and lack of motivation, played a decisive role in their participation in digital physical activity programs. These conditions, often accompanied by feelings of exclusion and social isolation, made participation even more difficult [51, 54, 56]. In addition, some participants highlighted that their desire to use technology was negatively affected by internalized negative beliefs about aging and environmental stigmatization. Moreover, participants mentioned that mild neurological disorders, such as cognitive decline, memory issues, attention deficit, and forgetfulness, which come with age, are major obstacles that limit their ability to keep up with these programs [54, 56].

*“I think you can quite easily get depressed when you’re really tired so you have to sort of shake your feathers and say oh come on get on with it.” (Maula et al.*,* 2019; ID6)* [54].

Many participants expressed that they were uncomfortable comparing daily measurement results, such as step counts, with their peers, felt embarrassed and afraid of failure, and even expressed disappointment that their social interaction expectations were not met because of the competitive environment [30, 57]. Several participants reported that not having a partner on virtual platforms was a barrier to social gaming. Some participants noted that when working in a group, they could only see the faces and shoulders of the individuals opposite them, and therefore experienced limitations in communication because they were unable to use non-verbal cues [50, 57].

*“When she arrived in one morning and I think she’d already done about five or six thousand steps*,* oh God*,* put me to shame*,* so I wouldn’t be able to compete with them like.” (O’Brien et al.*,* 2021; P2)* [57].

*“.Even reading kind of like subtle facial expressions*,* or*,* like those kinds of nonverbal responses*,* and that type of thing*,* obviously*,* can’t quite be figured out in the same way as an in-person scenario.” (Akinrolie et al. 2024; P2)* [50].

#### Individual and environmental limitations

Many participants related to the individual dimension reported negative experiences with technology due to reasons such as lack of motivation, difficulties with time management, lack of habits, limited physical space, and social isolation caused by the COVID-19 outbreak. However, they also highlighted that discomfort in mixed-gender groups and reservations stemming from social norms limited participation [47, 54]. Regarding the environmental dimension, most participants stated that their access to and continuity of digital physical activity applications were seriously affected by factors such as lack of suitable venues, high costs, inadequate transportation options, adverse weather conditions, class schedules that did not fit in with their daily lives, and physical venues that were inadequate in terms of safety, location, or accessibility [47, 54, 59]. These multi-layered barriers highlight the need for strategic planning that is sensitive to individual needs, considers socio-cultural differences, and considers environmental conditions.

*“You have got to regiment yourself to do these things haven’t you?” (Maula et al.*,* 2019; ID29)* [54].

*“I have a very small space*,* probably about 400 square feet. Not very big. For me it was really hard. They [the exercise staff] wanted to see me and I’m trying to exercise and I’m trying to hold my phone at the same time. That doesn’t work*,* because they want it at a certain level and want to be able to see you from head to toe while exercising.” (Dunston et al.*,* 2024; P66)* [47].

*“Yes I think the weather does*,* I think you know if it was pouring down with rain or something like that*,* I would probably be reluctant to go out and walk far in it.” (Maula et al.*,* 2019; ID11)* [54].

### ConQual summary

The ConQual summary indicates the confidence level of the results obtained by providing confidence ratings for each synthesized finding. As a result of the meta-aggregation process, two main synthesized finding were identified: (1) older adults’ pathways to meaningful technology use for physical activity and (2) structural and personal limitations in digital health engagement. According to the reliability assessment results of the included studies using five items in the JBI-QARI (Q2, Q3, Q4, Q6, Q7): one study scored 2/5 and seven studies scored 3/5, assigning them a medium reliability rating; six studies scored 4/5 and two studies scored 5/5, assigning them a high reliability rating. However, due to methodological quality concerns, the reliability levels of the synthesized findings were lowered by one grade. In addition, the credibility levels of the synthesized findings consist of a mixture of “Unequivocal” and “Equivocal” findings. Therefore, the credibility levels of the synthesized findings were lowered by one degree [42]. As a result, the overall JBI ConQual scores of the synthesized findings were rated as “moderate.” The process of confidence evaluation is detailed in Table 5.

**Table 5:** ConQual summary of findings (*n* = 183)

Synthesized finding	Design	Dependability	Credibility	ConQual score
Older adults’ pathways to meaningful technology use for physical activity	Qualitative	Downgrade 1 level^a^	Downgrade 1 level^b^	Moderate
Structural and personal limitations in digital health engagement	Qualitative	Downgrade 1 level^a^	Downgrade 1 level^b^	Moderate

*Abbreviations:a* Downgraded one level due to common dependability issues across the included primary studies, *b* Downgraded one level due to a mix of unequivocal and credible findings

## Discussion

A total of 16 qualitative studies were combined to enhance physical activity among older people. The findings highlight the importance of addressing individual, social, technological, and environmental factors in an integrated way. According to the aggregated results, technology can support older adults in engaging in physical activities; nevertheless, this is closely tied to considerations such as the suitability of the environment, social connections, motivational support, and ease of use. However, these experiences were found to be negatively impacted by environmental constraints, social isolation, physical and psychological impediments, and technology limitations. The significance of creating digital solutions that are accommodating, accessible, flexible, and supportive of the needs of senior citizens is highlighted by this.

### Synthesised findings 1: older adults’ pathways to meaningful technology use for physical activity

The ability of participants to adjust to technology has become a significant enabler of participation in digital physical activity programs; specifically, accessible, user-friendly, and configurable technologies have facilitated this process. It has been simpler for individuals to incorporate wearable technology and digital platforms into their daily lives because of their versatility, visual/auditory richness, usability, and adaptability to different lifestyles. When it comes to older people’s adoption of technology, ease of use and adaptability to their lifestyle are two of the most important elements [60]. Older people may be more inclined to embrace and incorporate healthcare technologies into their daily lives if the user interface is clear, easy to use, and visually simple [61]. Wearable technology, such motion tracking systems, smart bracelets, and pedometers, gives users immediate feedback, raising their level of self-awareness and favorably affecting their physical activity habits [62, 63]. Visual and aural richness are among the crucial aspects that boost technology participation, especially among persons with limited digital literacy [64]. Given these findings, it is imperative that digital physical activity programs created for older individuals be easy to use, adaptable, customizable, and suitable for their living situations in order to promote their adoption and long-term engagement with technology. These results can also be conceptually connected to models of technology acceptance, which highlight perceived utility and ease of use as key factors in technology adoption. The good experiences that older persons have described in this setting are consistent with theoretical frameworks that explain how and why people choose to use new technology, in addition to reflecting personal preferences. From an ecological point of view, accessibility, contextual relevance, and technological adaptability serve as environmental enablers that improve older persons’ digital engagement. When designing future intervention programs, user-centered approaches that take into account the talents, limits, and wider ecological contexts of older adults should be prioritized.

Participants reported feeling more internally satisfied and being motivated to increase physical activity as a result of technology-based interventions. Virtual success stories, wearable technology, quick feedback, and prizes have all been shown to raise people’s self-awareness and foster more enduring drive. The literature provides strong evidence for the conclusion that technology-based therapies can boost emotions of internal satisfaction and increase physical activity in a motivating way [27, 65, 66]. People can more efficiently monitor their activities thanks to these technologies, and the instant feedback loop serves to strengthen awareness and motivation [67]. Additionally, the incentives and virtual successes offered by these digital interventions boost users’ self-esteem, which promotes consistent participation in physical exercise regimens [10, 59]. Crucially, these results may be interpreted in a meaningful way using well-known behavioral and aging models, like the Ecological Models of Active Aging and the Technology Acceptance Model (TAM). Beyond merely enumerating facilitators and barriers, these frameworks provide conceptual integration that explains how perceived utility, simplicity of use, and compatibility with a person’s surroundings and lifestyle all support long-term adoption and ongoing motivation. The intrinsic motivation effects discovered are directly supported by TAM, which emphasizes the significance of intuitive and user-friendly design in enhancing perceived usability. The interaction between people and their larger social and technical contexts is also emphasized by ecological models, which highlight the importance of social feedback and customized goals in promoting long-term behavioral engagement in older adults. Therefore, it is crucial that technology-based physical activity programs for senior citizens include components that support psychological well-being and self-awareness while also fitting with theoretical frameworks that take into account the complex relationship between aging and behavior modification. Using this lens while designing interventions guarantees a more profound and long-lasting effect on motivation and long-term engagement.

Participants reported that telemedicine-based digital exercise programs effectively increased physical activity levels by strengthening social ties and mutual support systems through virtual interaction. The sustainability of physical activity and adherence to digital programs were positively impacted by collaborative technology use, technical and emotional support, and an increase in self-efficacy. Telemedicine-based digital exercise programs increase physical activity by strengthening social ties and mutual support systems through virtual interaction [68–70]. One of the main factors that motivates people to engage in physical activity, particularly older folks, is social support [71, 72]. These results demonstrate how digital platforms and telemedicine encourage older persons to engage in physical activity and stay motivated by fostering more social contacts. Participants’ self-confidence and dedication to the program are reinforced via group-based virtual activities and technical support systems. As a result, when digital interventions are combined with social support, older persons’ physical activity habits significantly and sustainably improve.

Exercise at home has offered benefits in terms of sustainability and comfort, and environmental considerations (location, safety, weather, financial status, and transportation) have been crucial in determining participation in digital physical activity programs. Maintaining privacy, having enough room in a comfortable and secure setting, and having the right environment have all been crucial in boosting participants’ dedication to the program. Evidence-based digital exercise interventions can be used in real-life home settings to improve the health of older individuals in the community. They have been demonstrated to increase older adults’ physical activity duration and participation [73]. Exercise at home promotes sustainability because it offers a comfortable, familiar, and safe setting [74, 75]. Because it offers a comfortable, familiar, and safe setting, it is stressed that working out at home promotes sustainability. Furthermore, elements like enough physical space and privacy make older persons feel at ease, which increases their dedication to the program [76]. In this context, the appropriateness of environmental elements is a key determinant of the efficacy and usability of digital interventions. These results imply that taking into account environmental facilitators could improve the success of applications related to digital health.

### Synthesised findings 2: structural and personal limitations in digital health engagement

Technical and functional obstacles, including complicated user interfaces, malfunctions, poor connectivity, and a lack of device customization, adversely affected their experiences engaging in digital and physical activities. Participation in such interventions was also decreased by privacy and security concerns as well as the requirement for technical assistance; participants stressed the value of device usage and data protection training. Complex user interfaces, technical issues, and poor connection quality are some of the difficulties older people encounter when utilizing digital technologies [77, 78]. Specifically, age-related cognitive and physical changes might negatively impact the user experience and make using these devices even more difficult [79]. Concerns regarding data security and privacy among participants align with the literature’s frequent emphasis on a lack of digital trust [75]. People who receive inadequate technical support services are left to handle problems on their own, which eventually makes them less interested in intervention. Furthermore, among people with poor levels of digital literacy, a lack of instruction on data protection and device usage reduces their incentive to participate [80, 81]. These results imply that the success of digital health applications depends on technological proficiency and trust in security-related matters. To guarantee wider participation, digital intervention projects should emphasize user-friendly design and incorporate standardized, easily accessible user training sessions. The policies should use public health tactics that provide fair access to training and support in order to advance digital health literacy. Health care systems should also be urged to incorporate digital support services in community settings in order to offer ongoing help. For strategies to be more effective over the long run, they must be incorporated into policy frameworks. This is especially true for older persons from socioeconomically disadvantaged backgrounds.

Computerized physical activity programs may occasionally result in detrimental emotional and bodily effects like weariness, pain, psychological distress, and social isolation. Furthermore, it has been noted that a number of variables make technology engagement and sustainability more difficult, including age-related cognitive loss, negative beliefs, technical shortcomings, and feelings of humiliation in competitive contexts. Some older persons may experience negative effects from participating in digital programs, including weariness, musculoskeletal pain, and a decline in motivation [82]. Nonetheless, common personal obstacles that prevent the use of digital programs include cognitive deterioration and unfavorable attitudes toward technology [83]. Particularly for people with poor self-esteem, the social isolation and embarrassment felt on competitive platforms can lead to psychological stress [84]. Additionally, using digital tools is more stressful and time-consuming due to age-related learning challenges and technical shortcomings [85]. These instances show the dangers of using digital treatments without considering each person’s unique requirements and features [86]. Therefore, creating customized digital physical exercise programs that take into account the mental, emotional, and physical well-being of senior citizens is crucial. These insights ought to guide the creation of policies that require mental and emotional health evaluations to be included in the process of developing programs. In order to identify those who are at risk of negative consequences and to customize interventions appropriately, health care providers may be extremely important. Reducing digital disparities also entails acknowledging the emotional aspects of digital participation, which must be taken into account when developing inclusive initiatives, in addition to granting access.

Individual and sociocultural factors, including social isolation, time management challenges, lack of motivation, physical space constraints, and social norm concerns, were found to have a detrimental impact on participants’ engagement in digital physical exercise programs. Additionally, the accessibility and sustainability of these programs have been restricted by external factors like a lack of appropriate venues, exorbitant costs, transportation challenges, and environmental conditions. This underscores the necessity of strategic approaches that are considerate of environmental barriers and sensitive to individual differences. The literature highlights how personal issues including social isolation, time management challenges, and a lack of motivation directly affect older persons’ ability to engage in physical activity [87, 88]. People’s motivation to engage might also be lowered by worries about sociocultural norms, such as the lack of physical space and how society views age [89]. Transportation, space constraints, and cost are examples of external issues that make access difficult, which negatively affects the long-term sustainability of digital programs [90]. These intricate restrictions imply that complete social and environmental infrastructures are necessary to enable interventions. It takes adaptable models that take into consideration both environmental access and human capabilities to create digital physical activity programs that work. These findings, taken as a whole, urge governmental measures that lower structural obstacles, such as subsidizing access to digital tools, establishing secure public areas for physical activity, and incorporating technology-enabled health initiatives into community centers. To guarantee that these services are accessible and suitable for the local culture, health systems must work with local authorities. Accordingly, addressing socioeconomic and geographic inequalities that affect older individuals’ capacity to access and maintain digital participation is also a necessary part of minimizing digital inequalities.

The 16 studies included in this meta-synthesis were conducted in eight different countries (the United States, Canada, Australia, the United Kingdom, Japan, Germany, Ireland, and Sweden) and within diverse socio-cultural and technological contexts. The various differences reflected in participant experiences are associated with levels of digitalization across countries, older individuals’ access to technology, social values, and the organizational structures of healthcare services. For example, in some countries (e.g., USA, Canada), older individuals were found to be more familiar with technology and to have greater access to technical support, whereas in other countries (e.g., Japan, Germany), stronger privacy concerns and cultural reservations were observed. The technologies employed also vary considerably; for instance, some studies utilized virtual reality games, wearable trackers, or voice assistants (such as Alexa), whereas others featured telehealth applications or platforms focused on social support. However, older adults’ adaptation to technology and their levels of satisfaction were influenced by factors such as the context in which the technology was used, the technical support provided, individuals’ living conditions, socioeconomic status, and social norms. These findings underscore the need for technology-based physical activity interventions to be designed in a manner that is sensitive to the sociocultural context in which they are implemented, and highlight the importance of developing context-specific strategies rather than universal solutions.

### Limitations and strengths

This meta-synthesis has some limitations. First, while the review process used a comprehensive search approach across four major databases (PubMed, Scopus, CINAHL, and MEDLINE), it did not include grey literature. Grey literature was excluded because it does not consist of high-level peer-reviewed publications. Second, only English-language studies were considered; results from publications in other languages in this field could not be included in this meta-synthesis. Another important limitation is that half of the studies included in the meta-synthesis had a moderate risk of bias despite the systematic use of the JBI-QARI method for quality rating. The high prevalance of moderate risk of bias generally related to the researcher’s cultural position in the study and the lack of explanation of the researcher’s influence on the study. Qualitative research may also involve subjective analyses that are susceptible to researcher biases.

In spite of these limitations, the study has several strengths. This meta-synthesis was carried out in accordance with accepted practices, including the JBI Manual for Evidence Synthesis, JBI-QARI, ConQual approach, ENTREQ, PRISMA, and SPIDER with methodological rigor and transparency. Nonetheless, it is necessary to recognize a number of restrictions. The findings are more reliable when a strong and open approach is used, which includes double-blind screening, independent data extraction, critical evaluation, and consensus-building procedures. The results’ transferability is enhanced by the inclusion of studies from various geographical locations and contexts. Furthermore, the JBI meta-aggregation method’s use made it possible to conduct a systematic synthesis that yields useful results while honoring the original meanings of the main investigations. Finally, the results’ credibility and relevance for researchers, practitioners, and policymakers interested in using technology to encourage physical activity among older individuals are reinforced by the application of the ConQual framework to gauge confidence in synthesized findings. In this study, although the screening was conducted without a year restriction, only studies from recent years were included in the meta-synthesis due to the contemporary nature of the topic. Therefore, since the studies included in the meta-synthesis had undergone rigorous peer review, their methodological quality was found to be high or moderate.

## Conclusion

This study has uncovered the complex elements that both support and impede older adults’ engagement in technology-based therapies meant to boost their levels of physical activity. Participation is boosted by social support, environmental suitability, incentive elements, ease of use, and customisation. On the other hand, a lack of personal motivation, social isolation, environmental limitations, physical and mental health problems, and technical limitations were shown to be major obstacles to participation. It is important to create user-friendly interfaces and designs that may be altered to suit individual needs in order to improve participants’ technological adaptability. In order to minimize technological concerns, ongoing technical assistance and privacy awareness training should be offered. Supporting group projects and family involvement that promote social contact is necessary to guarantee the durability of initiatives. Plans should also be made for safe activity that reduces hazards based on physical fitness levels. Adopting customized strategies that consider environmental factors and sociocultural variations is recommended. Solutions that lower physical and transportation obstacles should be created, and digital applications should be made more economically accessible. Multifaceted strategic planning is necessary to ensure the success and inclusivity of technology-based physical activity programs.

## Supplementary Information

Supplementary Material 1.

Supplementary Material 2.

## Data Availability

All data generated or analysed during this study are included in this published article [and its supplementary information files].

## Abbreviations

JBI: Joanna Briggs Institute
JBI-QARI: Joanna Briggs Institute Critical Appraisal Checklist for Qualitative Research
ENTREQ: Enhancing Transparency in Reporting the Synthesis of Qualitative Research
PRISMA: Preferred Reporting Items for Systematic Reviews and Meta-Analyses
SPIDER: Sample, Phenomenon of Interest, Design, Evaluation, Research type
