# Supplementary material for: Experiences of technology for increasing physical activity of older adults: a qualitative systematic review and meta-synthesis
Source: Eur Rev Aging Phys Act. 2025 Dec 5;23:2. doi: 10.1186/s11556-025-00394-7 (PMC12797423; doi:10.1186/s11556-025-00394-7)
Supplement: Supplementary file 1 — Supplementary Material 1. [file 11556_2025_394_MOESM1_ESM.docx]

**Additional file 1** The detailed search strategy conducted in PubMed, Scopus, CINAHL and MEDLINE databases (last search date: January 28, 2025)

| **Databese** | **Search** | **Concept** | **Key Terms** | **Search results** | **Total number of results*** |
| --- | --- | --- | --- | --- | --- |
| PubMed | #1 | older adult | ((((((("older adult"[Title/Abstract]) OR ("older people"[Title/Abstract])) OR ("elderly"[Title/Abstract])) OR ("aged"[Title/Abstract])) OR ("geriatric"[Title/Abstract])) OR ("aging"[Title/Abstract])) OR ("ageing"[Title/Abstract])) OR ("senior"[Title/Abstract]) | 1,472,529 | 144 |
|  | #2 | physical activity | (("physical performance"[Title/Abstract]) OR ("exercise"[Title/Abstract])) OR ("physical activity"[Title/Abstract]) | 505,807 |  |
|  | #3 | technology | ((((("digital*"[Title/Abstract]) OR ("technolog*"[Title/Abstract])) OR ("electronic"[Title/Abstract])) OR ("telehealth"[Title/Abstract])) OR ("wearable device"[Title/Abstract])) OR ("telemonitoring"[Title/Abstract]) | 1,441,047 |  |
|  | #4 | qualitative | (("qualitative research"[Title/Abstract]) OR ("qualitative study"[Title/Abstract])) OR ("qualitative methods"[Title/Abstract]) | 121,013 |  |
| Scopus | #1 | older adult | ( TITLE-ABS-KEY ( "older adult" ) OR TITLE-ABS-KEY ( "older people" ) OR TITLE-ABS-KEY ( "elderly" ) OR TITLE-ABS-KEY ( "aged" ) OR TITLE-ABS-KEY ( "geriatric" ) OR TITLE-ABS-KEY ( "aging" ) OR TITLE-ABS-KEY ( "ageing" ) OR TITLE-ABS-KEY ( "senior" ) ) | 8,044,647 | 746 |
|  | #2 | physical activity | ( TITLE-ABS-KEY ( "physical performance" ) OR TITLE-ABS-KEY ( "exercise" ) OR TITLE-ABS-KEY ( "physical activity" ) ) | 1,098,029 |  |
|  | #3 | technology | ( TITLE-ABS-KEY ( "digital*" ) OR TITLE-ABS-KEY ( "technolog*" ) OR TITLE-ABS-KEY ( "electronic" ) OR TITLE-ABS-KEY ( "telehealth" ) OR TITLE-ABS-KEY ( "wearable device" ) OR TITLE-ABS-KEY ( "telemonitoring" ) ) | 8,254,858 |  |
|  | #4 | qualitative | ( TITLE-ABS-KEY ( "qualitative research" ) OR TITLE-ABS-KEY ( "qualitative study" ) OR TITLE-ABS-KEY ( "qualitative methods" ) ) | 336,003 |  |
| EBSCO – CINAHL Ultimate | #1 | older adult | XB “older adult” OR XB “older people” OR XB “elderly” OR XB “aged” OR XB “geriatric” OR XB “aging” OR XB “ageing” OR XB “senior” | 477.293 | 42 |
|  | #2 | physical activity | XB "physical performance" OR XB "exercise" OR XB "physical activity" | 287.655 |  |
|  | #3 | technology | XB "digital*" OR XB "technolog*" OR XB "electronic" OR XB "telehealth" OR XB "wearable device" OR XB "telemonitoring" | 308.479 |  |
|  | #4 | qualitative | XB "qualitative research" OR XB "qualitative study" OR XB "qualitative methods" | 3.933 |  |
| EBSCO  HOST- MEDLINE | #1 | older adult | XB “older adult” OR XB “older people” OR XB “elderly” OR XB “aged” OR XB “geriatric” OR XB “aging” OR XB “ageing” OR XB “senior” | 1.050.301 | 109 |
|  | #2 | physical activity | XB "physical performance" OR XB "exercise" OR XB "physical activity" | 287.655 |  |
|  | #3 | technology | XB "digital*" OR XB "technolog*" OR XB "electronic" OR XB "telehealth" OR XB "wearable device" OR XB "telemonitoring" | 308.479 |  |
|  | #4 | qualitative | XB "qualitative research" OR XB "qualitative study" OR XB "qualitative methods" | 9.915 |  |

*Abbreviations:* * #1 AND #2 AND #3 AND #4; TITLE-ABS-KEY: Title-Abstract-Keywords; XB: Title and Abstract
