# Supplementary material for: Experiences of technology for increasing physical activity of older adults: a qualitative systematic review and meta-synthesis
Source: Eur Rev Aging Phys Act. 2025 Dec 5;23:2. doi: 10.1186/s11556-025-00394-7 (PMC12797423; doi:10.1186/s11556-025-00394-7)
Supplement: Supplementary file 2 — Supplementary Material 2. [file 11556_2025_394_MOESM2_ESM.docx]

**Additional file 2** The credibility assessment process for illustrative quotes related to synthesized findings and categories (n=371)

| **Synthesised findings 1. Older adults’ pathways to meaningful technology use for physical activity** | **Illustrative Quotes** | **Credibility** |
| --- | --- | --- |
| Category 1. Adaptation to technology and ease of use | *“I feel like I’m facing real life, but the design is not quiet. I want to be immersed more in the game.” (P9) (Hosseini et al., 2024)* | U |
|  | *“The functions on the smartwatch were very clear… Charging was no problem. And then just swiping it and finding the information was very easy.” (P7) (Li et al., 2024)* | U |
|  | *“The face was very easy to read for a person who’s just been diagnosed with age-related macular degeneration” (Study 1 Participant #3) Li et al., 2024* | U |
|  | *"a good way to exercise" (P9) (Hosseini et al., 2024)* | C |
|  | *“And I’m in my little exercise room now. I had… this condo was remodeled so I made sure I had certain things built in so I didn’t have to stick those bands in the door and then have them come flying at me. [Everyone laughs] We’ve all experienced that one!” (F, 67) (Dunston et al.,2024)* | U |
|  | *“I liked that the program was personalized to me. They tried to work with things I had problems with. For example: I have a really bad right knee that I should have replaced and because of the cancer diagnosis I was not able to do the knee replacement, and so they tried to work with me to strengthen things but work within my abilities.” (F, 66) (Dunston et al., 2024)* | U |
|  | *“Plus, again as the previous caller mentioned, you can do it anywhere in the world and that is very convenient” (M, 69) (Dunston et al., 2024)* | U |
|  | “Well, it is just that it’s really easy. You don’t have to leave your house. You don’t even have to get out of your pajamas if you don’t want to. It’s really easy. I liked it. . .” (older adult, female, 65 years) (Akinrolie et al., 2024) | U |
|  | “I think the good parts of it are, like I said, it’s more flexible, both for myself and for the participants [older adults] So, I can take the meeting anywhere I am, they can take the meeting anywhere they are. . .” (counsellor 1). (Akinrolie et al., 2024) | U |
|  | “The nice thing about zoom actually is that I was able to have the file [the counselling guide] open on the side without the client knowing so then I can still kind of try to take a glance at it” (counsellor 3) (Akinrolie et al., 2024) | U |
|  | *“Yeah, I [began to use a WAM because I] wanted to make sure I’m really walking enough, because I thought I was, but this way I really can make sure I’m doing at least two miles every day.” —Female, ever WAM user, “more active.” (Zytnick et al., 2023)* | U |
|  | *“I mostly used the inactivity tracker, where it tells me to get off the computer and move.” –Female, ever WAM user, “less active.” (Zytnick et al., 2023)* | U |
|  | *“At least the Fitbit^TM^ was pretty menu-driven. Once you get the app set up on your phone, it takes you through step by step.” –Male, ever WAM user, “more active.” (Zytnick et al., 2023)* | U |
|  | *“I treated myself last summer to the ventilated band in a pretty color, which is a little bit cooler” –Female, ever WAM user, “less active.” (Zytnick et al., 2023)* | U |
|  | *‘I hadn’t used Zoom before but I can do it now, I’m glad I learned how to do this during the program’ (p41/M/online) (Weselman et al., 2023)* | U |
|  | *“Trick is to make it sufficiently interesting and challenging to those who find it fairly easy, but also not to put off people who find it harder and struggle to get out of the chair, so maybe if you had a series of levels so you could come in at level one or you could jump to level three.” (P3) (Stawarz et al., 2023)* | U |
|  | *One resident who switched hands after beating the game with their dominant hand reported that the exergame made them “much more aware of [their] motion.”  (Crane et al., 2023)* | U |
|  | *“I particularly like the voice thing if you can just say it without having to go and sort of manually touch the screen, yeah. That’s good.” (Participant 14, male, age 74) (Jansons et al., 2022)* | U |
|  | *“Yeah no, it’s good. You don’t have to touch it.” (Participant 9, female, age 65). (Jansons et al., 2022)* | U |
|  | *“A phone would probably do something similar…, but I mean it’s still you have to then go and dive into the app and all that sort of stuff, I guess.” (Participant 14, male, age 74). (Jansons et al., 2022)* | U |
|  | *“Oh yes, I didn’t need to be close to it to do the exercises. I could do it from a distance and it responded to my answers.” (Participant 8, female, age 67). (Jansons et al., 2022)* | U |
|  | *“Anyway, it can stay where it is, you know what I mean? I don’t have to transport it around the house and the volume is important that you can adjust the volume. It is kind of like a more-or-less permanent fixture in the house. So that was good- it was inobtrusive and small enough but usable enough if you know what I mean.” (Participant 3, female, age 67). (Jansons et al., 2022)* | U |
|  | *“The positive is it’s there, you can use it as many times as you want, it’s handy, it’s close by.” (Participant 1, male, age 70). (Jansons et al., 2022)* | U |
|  | *“It’s easy to use. There is a lot of exercises. You can skip what you don’t want. You do what you want and what you need. Those are all pluses.” (Participant 1, male, age 70). (Jansons et al., 2022)* | U |
|  | *“I enjoyed using it. I found Alexa (and the Buddy Link software program) very easy to use.” (Participant 7, female, age 67). (Jansons et al., 2022)* | U |
|  | *“Yeah. I think I might’ve mentioned that such a program could probably be implemented just as easily and alternatively as a web-based solution, which would just avoid all the annoyance of the voice-based program.” (Participant 6, male, age 77). (Jansons et al., 2022)* | U |
|  | *“I've got my old phone, my present phone, an old tablet, on a really old tablet as well, and plus my laptop. I think it's a much better idea to put it onto the web, honestly.” (Participant 11, male, age 71). (Jansons et al., 2022)* | U |
|  | “Because it’s a bigger screen. And it’s more comfortable. No, I’d always use the laptop.” – P11, female, age 74 (Simmich et al., 2021) | U |
|  | “I could not do without my computer nowadays, particularly for emails.” – P1, female, age 66 (Simmich et al., 2021) | U |
|  | *“No, I had no difficulties with it, […] I’m not a techy kind of a person, you know? But I, I was able to set the time on it when I went walking and I was able to look and see what my heart rate was and […] How many steps I had done and stuff, you know I didn’t have a problem with it.” (P11) (O’Brien et al., 2021)* | U |
|  | *“Well the benefit was I used to think I was a very bad sleeper, but now I realise actually I get quite a lot.” (P2) (O’Brien et al., 2021)* | U |
|  | *“I also put an alert on my [tracking device] to, you know it beeps three times if I’ve been sitting down for an hour. So, that makes me get up and do a few jumps or whatever you know? […] and I found that was a good part of it as well.” (P8) (O’Brien et al., 2021)* | U |
|  | *“Monitoring the number of steps with the Fitbit is informative.” (participant, female, ≥70 years, high level of education) (Wichmann et al., 2020)* | U |
|  | “My fitbit allowed me to personalize my exercise. I learned new things about myself from the fitbit” (4B). (Abouzahra and Ghasemaghaei 2020) | U |
|  | *“Fitbit helped me a lot. It told me when it was time to exercise and I would get up and walk around the house or take my dog out”. (1B) (Abouzahra and Ghasemaghaei 2020)* | U |
| Category 2. Motivation and intrinsic/personal satisfaction | *“I found it exciting!<LAUGHS> And I always wanted to do better, beat my score, or try to get to that next level. So it kept me coming.” (Crane et al., 2023)* | U |
|  | *“a pleasant experience” (Study 2 Participant #5) (Li et al., 2024)* | C |
|  | *“It was attractive” (Study 2 Participant #2) (Li et al., 2024)* | C |
|  | *“I am amazed how steps accumulate on the smartwatch…I’ve been hooked” (Study 1 Participant #7); (Li et al., 2024)* | U |
|  | *“It is very interesting to see how the step counts go up while you walk” (Study 2 Participant #2) (Li et al., 2024)* | U |
|  | *“When I look at my-- I have done 3,649. I have 351 more steps to do before I get to my goal, and I’m going to do it” (Study 3 Participant #8) (Li et al., 2024)* | U |
|  | *“It’s just very nice. You know where you are. It’s nice because you feel like you are making progress, and you have little steps. You know how much effort it needs to get there” (Study 3 Participant #1) (Li et al., 2024)* | U |
|  | *“When I wanted to get up from a very low number of steps, I would take a walk after dinner or in the late afternoon. So I would make a conscious effort to try to reach the goal” (Study 1 Participant #6) (Li et al., 2024)* | U |
|  | *"*I felt so excited*!*It was fun*!*It’s really neat*!*It just brings happiness" (P5)*. (Hosseini et al., 2024)* | U |
|  | "It’s been a fun experience which I am thankful for" (P11). (Hosseini et al., 2024) | U |
|  | "Neat*,*awesome"*(P14) (Hosseini et al., 2024)* | C |
|  | "I liked the environment; it was very beautiful, the underwater environment" (P11) (Hosseini et al., 2024) | U |
|  | “Something that I found interesting was in the background. The animals and the other things going on and watching the big cat, the lion…" (P6) (Hosseini et al., 2024) | U |
|  | "How crazy is that in this world? You are not vulnerable even, the VR, move a piece, and it happens right away!" (P5). (Hosseini et al., 2024) | U |
|  | "It was very engaging… it built intensity… it wasn’t just one thing around every minute. It slowly introduced maneuvers, picked up the speed, and became very challenging" (P4). (Hosseini et al., 2024) | U |
|  | *"*I love music; I love dancing*,*so then it brought more of me with the music*.*The music*,*I would say*,*I’m present more*.*[Laugh]"*(P7). (Hosseini et al., 2024)* | U |
|  | “I think the challenge of getting as highest score as you possibly can, so you do it once, you get a score, the second time you want to beat your score.” (P4) (Hosseini et al., 2024) | U |
|  | “It takes me out of my everyday tasks and everything and brings me to another world… you see the fishes and things like that and makes you forget you are in the house, and you take the thing [headset] out, and you see oh! I’m in the house" (P12) (Hosseini et al., 2024) | U |
|  | “A really good space to be in, after you have a bad day, wow! Increase your thoughts and your endorphins, you know, just puts you in a happy mood.” (P5) (Hosseini et al., 2024) | U |
|  | *“One more thing I liked about it is I knew every Monday and every Wednesday I had to be up at a certain time and be in there doing the exercises and they would ask me about my outside exercise and that provided a little additional stimulant to get my outside exercises performed as well.” (M, 71) (Dunston et al., 2024)* | U |
|  | “I’m walking. . .. I’m going to try to walk three times a week at least for half an hour” (Older adult, male, 72 years) (Akinrolie et al., 2024) | U |
|  | “I think I was more aware. And I said to myself, come on now you’ve got to move. You got to get out there and go for a walk in the morning when I woke up from the bed. I was thinking maybe I will go to the pool today. Oh no better go. . ..” (Older adult, female, 65 years) (Akinrolie et al., 2024) | U |
|  | “. . .Oh, 10 [level of confidence], yeah. Absolutely I have no lack of confidence. Thankfully, I will capitalize on the fact that I still have good health and I have still mobility” (Older adult, female, 69 years) (Akinrolie et al., 2024) | U |
|  | *“If I didn’t have the goal and the number, I would just sit and watch television, but I want to meet my goal. It’s a strange psychology.” —Male, ever WAM user, “less active.” (Zytnick et al., 2023)* | U |
|  | *“I’m walking more, because I know at five miles fireworks go off [on my WAM and…] I realize internally I love it.”–Female, ever WAM user, “less active.” (Zytnick et al., 2023)* | U |
|  | *“[I am] addicted to [WAM]” because “I started with the losing weight, and I just…keep pushing myself to do more and more steps.” –Female, ever WAM user, “less active.” (Zytnick et al., 2023)* | U |
|  | *“If you spend the money to have it, to me, that makes me want to use it.” –Female, ever WAM user, “more active.” (Zytnick et al., 2023)* | U |
|  | *“I think that’s a motivator, I need to have something I can do that’s close to my doorstep :::” (p7/F/in person) (Weselman et al., 2023)* | U |
|  | *“ ::: my family has a history of heart problems, so I want to keep as active as I can” (p4/F/in person). (Weselman et al., 2023)* | U |
|  | *“I am trying to change that old mind-set of being so negative and re-phrase it to a positive, so not be so hard on myself,U*  *and remind myself anything is better than no exercise” (p14/F/in person). (Weselman et al., 2023)* | U |
|  | *“I was already a member before the program, but I increased my membership to Gold, so I could attend more activities during the week” (p8/F/in person). (Weselman et al., 2023)* | U |
|  | *“it gave me the inspiration to go and look for what I wanted in other groups and clubs that I could join” (p7/F/in person). (Weselman et al., 2023)* | U |
|  | *“ ::: gave education and tips on how to live better::: making me more aware of how important it was to keep doing these activities, even when you don’t feel like it” (p18/M/in person). (Weselman et al., 2023)* | U |
|  | *“I learned that it’s okay to do a bit more exercise than I normally would have done” (p17/F/in person). (Weselman et al., 2023)* | U |
|  | *“If it’s there, you’ll use it. And that’s just getting into the regime, it’s like, in the morning, you'll sort of do 10 minutes of different exercises...And it just becomes a habit.” (P4) (Stawarz et al., 2023)* | U |
|  | *“The little pad would be sitting on the floor, would be a reminder.” (P5) (Stawarz et al., 2023)* | U |
|  | *“At the moment I do it when I can see I’ve got two or three minutes to do sit to stand. So I do.” (P1) (Stawarz et al., 2023)* | U |
|  | *“When members provide a topic, such as a flower series or a cooking series, I want to get on it, but I have not done any cooking to take a photo... I know that daily meals are fine, but there is just something about posting them that makes me feel self-conscious.” (Participant B) (Shinokawa et al., 2023)* | U |
|  | *“I go to play table tennis as soon as the gymnasium opens at 8:30 am. I wish I could take a picture that would make everyone thinks “How can an older person like them (participant H) do this?” (Participant H) (Shinokawa et al., 2023)* | U |
|  | *“I’m glad that everyone praised my step count.” (Participant H) (Shinokawa et al., 2023)* | U |
|  | *“I feel lonely when I don’t get a reaction to my post.” (Participant A) (Shinokawa et al., 2023)* | U |
|  | *“They are doing what I aim for. If I think they’re doing great at something, I want to make an effort to get as close as possible to it.” (Participant H) (Shinokawa et al., 2023)* | U |
|  | *“I've been through a doctoral study, and when [one of the researchers] said she might be involved in this as part of her degree, I had no problem setting my foot with it because I've been there, done that. And if I can help, then I was happy to help.” (Crane et al., 2023)* | U |
|  | *One participant joined “to improve my motions and mind activity together.” (Crane et al., 2023)* | C |
|  | *Participants also enrolled because they were curious about the unique nature of the exergame and “thought it would be something interesting to do. Because sometimes it gets kind of boring. And it was something different.”  (Crane et al., 2023)* | C |
|  | *“The hour sped by. And there was no boredom on my part at all, because you, again, were looking to better your score, and either that or get to the next level.” (Crane et al., 2023)* | U |
|  | *“I don't think boredom is one of the issues at all. Because… the time went very quickly.” (Crane et al., 2023)* | U |
|  | *“The exercise was a plus, because I found myself moving, as they say, moving and grooving <LAUGHS>, trying to, you know, reach that goal of getting that higher, getting to that next level.” (Crane et al., 2023)* | U |
|  | *“I look[ed] forward to my time, and I'm not a morning person, but I got myself up, dressed, and ready to go! Any other time, huh! I'd be in my nightclothes up until 12 o'clock if I didn't have anywhere to go.” (Crane et al., 2023)* | U |
|  | *“[It] was a challenge at times but overall, I looked forward to coming to every session and continuing my progress in the game”  (Crane et al., 2023)* | U |
|  | *“Oh, yeah… [it] just helped me make some connections in my brain that had gotten lazy, shall we say?” Crane et al., 2023* | U |
|  | *“Once you're able to win on a pretty regular basis, it becomes fairly routine.” Crane et al., 2023* | U |
|  | *“There were days when I thought, I'm never going to get through this session today… but all of a sudden I did, and so I was happy about that.” (Crane et al., 2023)* | U |
|  | *“It just was a real opportunity to actually play a video game, and to have a chance, see how far I could get. It was an enjoyable thing to do. I wasn't going to quit it.”  (Crane et al., 2023)* | U |
|  | *All men cited an obligation to fulfill commitments as a motivator for retention, stating: “A promise is a promise. So I stayed in it until it was over.” and “You signed up for this much duty, you do it.” (Crane et al., 2023)* | C |
|  | *“(An exercise program is difficult) to do… because sometimes you get lazy and you don’t really want to do things. But if you’re getting directions and instructions (from the Alexa) along the way, you’re doing it without an effort because it’s gentle.” (Participant 9, female, age 67). (Jansons et al., 2022)* | U |
|  | *“No. I think the thought behind it is good and I can only speak personally for myself that it made me more interested and motivated to do (the exercise program). Because if you’re by yourself all the time and you're not mixing with other people, or in a group situation, and I used to go to group exercises, to keep yourself motivated to do exercises several times a day when Uyou get to the age range that I'm in” (Participant 5, female, age 79). (Jansons et al., 2022)* | U |
|  | *“I did the exercise because she (Alexa) always asks me (do you) want to do it again? So, I did it again, but all the other times when I said I’ll do it again it’s because I was not happy with myself and I wanted to improve it. It’s a really thought through program” (Participant 3, female, age 67). (Jansons et al., 2022)* | U |
|  | *“The advantage of having it there, knowing that it was going to remind me to do my exercises was a good thing, because I do have a physio app on my phone, but I have to actually go onto it to use it, where this actually would be reminding me. I feel very guilty if I didn’t do it.” (Participant 9, female, age 65). (Jansons et al., 2022)* | U |
|  | *“I thought it was a great way to be reminded and would definitely influence me to exercise more. So I thought it was a very good thing from that point of view.” (Participant 7, female, age 67). (Jansons et al., 2022)* | U |
|  | *“Yeah, I found that it actually made you commit to doing it. Because it would give you reminders, you kind of felt obligated to complete it, you wouldn't just be going …Oh, I'm too busy,…or I can't be bothered….So I found that was really good. It sort of made you commit to it.” (Participant 2, female, age 66). (Jansons et al., 2022)* | U |
|  | “I’m not doing too bad for my age. A lot of guys my age don't even know…I've got a buddy, he doesn't even know how to a start up a phone.” – P15, male, age 67 (Simmich et al., 2021) | U |
|  | “When I first got it [Fitbit], I was doing about 3000 paces a day. Well, now my average is 4700. And that's only within the twelve-month period.” – P18, male, age 59 (Simmich et al., 2021) | U |
|  | “I’d benefit, because then I can see how much I’m doing and how far I’m going. And each day, or every couple of days, it should be a bit further and further.” – P4, female, age 74 (Simmich et al., 2021) | U |
|  | “I do want to clarify it a little bit more, and put it into more of a goal-setting and goal achievement type structure. And that's where, yeah, a bit of technology would help, like the Fitbit.” – P7, female, age 70 (Simmich et al., 2021) | U |
|  | “It was a motivator. One of my wife’s friends said, ‘I’ll get it for [P18], because it gives him a target, he’ll have to beat it.’ Which I did. I’ll not go to bed until I’ve done the four-thousand paces a day. And, yeah, I’m trying to get the average up to five thousand.” – P18, male, age 59 (Simmich et al., 2021) | U |
|  | “This [Apple Watch] here, if I sit for an hour it’ll go ‘tap tap tap’ on my wrist and say ‘get up’. […] It reminds me what I’m doing, you know, that’s probably the best.” – P5, male, age 70 (Simmich et al., 2021) | U |
|  | “I’ve got [a Fitbit] on, yes. It was given to me by my daughter-in-law. And I use that if you’re sitting…before we’ve finished our conversation, it’ll be asking me to take it for a stroll. It sends a little message if you’re still for an hour, like if you’ve been sitting, it will just remind you to get up and move. […] So that’s how I use that anyway. And yes, I’m quite happy with it really.” – P14, female, age 66 (Simmich et al., 2021) | U |
|  | “I can see it being a benefit, because it gives me a yard stick to work to, you know what I mean? When I come home, I’d have a look at it, I’d write it in the calendar or I’d have a book I keep. So I know that I’m progressing or I’m not. So that [Fitbit] thing, even though I don’t particularly like them, I can see where that would be a benefit to me. I can write down how much I did on Monday and how much I did on Tuesday. And it gives me something to work against.” – P17, male, age 73 (Simmich et al., 2021) | U |
|  | “Well they know I’m exercising. I’m not saying that I’m walking down to [the park], they probably think ‘Oh yeah…’, you know. At least if I have the watch and go to my doctor they know I am exercising.” – P19, female, age 82 (Simmich et al., 2021) | U |
|  | “I am quite likely to cheat and lie, if I could. But if I can’t get away with it, it would really be very good. I’d like to have a big brother looking over the shoulder at me and saying, ‘Hey, come on, you're not doing what you said you’re supposed to do or we told you to do’.” – P11, female, age 74 (Simmich et al., 2021) | U |
|  | “My husband used to like [Mario Kart] […] I’m not very good at it. I crashed all the time […] It affected my motivation. Absolutely. Because I think, ‘Mmm, I’m not very good at it’. I’d really like to be able to do it, you know. I’m probably more likely to try something by myself. Maybe I just need to, sort of, feel more comfortable doing something before I, you know, play with other people.” – P14, female, age 66 (Simmich et al., 2021) | U |
|  | “I like competitive…yeah, I find that if you go out and just play golf, but if you play in a competition, it’s the competitiveness that makes you try harder.” – P2, female, age 76 (Simmich et al., 2021) | U |
|  | “I want to be a contest. I don’t like it if you’re not contesting something. You don’t have to win but you do have to not embarrass yourself.” – P11, female, age 74 (Simmich et al., 2021) | U |
|  | “If I’m going to play a game, I want to do it for the enjoyment I get and the fun that it is. I don’t think that it has anything to do with anybody else or ranking me in any way. I don't think that would be helpful to me as a person.” – P3, female, age 76 (Simmich et al., 2021) | U |
|  | “While I’m waiting [at] the doctor, I usually sit there and play solitaire if they’ve got no magazines to read. […] If there’s nothing else to do, I’ll play solitaire.” – P10, male, age 62 (Simmich et al., 2021) | U |
|  | “Oh, I just think they [AVGs] would be more fun and you’d be moving more. Would be better for your health, I would imagine” – P15, male, age 67 (Simmich et al., 2021) | U |
|  | *“I was surprised at, you know I would have thought that I was completing ten thousa-in excess of ten thousand steps every day but it just shows you that it, I wasn’t, so it, it’s a good motivator in that way […] and it would push you on.” (P1) (O’Brien et al., 2021)* | U |
|  | *“Yeah just observing my own activity and ehm, becoming more self-aware of how active or inactive I am…I found myself pushing myself a little bit […] so it kind of made me more aware of building up my own stamina.” (P11) (O’Brien et al., 2021)* | U |
|  | *“I was watching it and doing my steps and it did encourage me to walk further, park further away in the car park and you know it encouraged me to build up the steps and […] I’d do a bit of walking, bit of exercise to get to a certain figure and so it was very encouraging from that point of view.” (P4) (O’Brien et al., 2021)* | U |
|  | *“In town, I always used to, I stopped using the escalator and I use the stairs. And I still use the stairs now in the shop, I don’t use the escalator anymore so, I thought if I only got just that one thing from it, it was probably worth doing.” (P4) (O’Brien et al., 2021)* | U |
|  | *“Now that I’d know if I wasn’t after doing enough of steps, you know I’d try and do something else then to catch up on it, you know.” (P10) (O’Brien et al., 2021)* | U |
|  | *“When I hadn’t that many steps done, I looked at it as, as something that made me kind of, encouraged me to do something which was for my good and for the good of everybody else as well like, you know? I mean if, if I’m healthy it keeps me out of hospital, leaves more, more space for other people you know that kind of thing, you know?” (P6) (O’Brien et al., 2021)* | U |
|  | *“I think you don’t need these things to eh, motivate you to get out to the beautiful country side, go for a walk with your dogs, or whoever and I’m all in favour of eh, you know, getting motivation to keep fit and all of that, but I am [70+] and I think that I, I change my way say maybe for a week or two and I say this is great, this is wonderful and then I say, throw it all up in the air.” (P7) (O’Brien et al., 2021)* | U |
|  | *“I thought that we’d be walking as a group […], I was hoping like that, you, I’d be motivated because I’d have to start at a particular time and meet people and do it that way, you know? […] the [tracking device] itself didn’t do anything for me” (P5) (O’Brien et al., 2021)* | U |
|  | *“It would be something I’d do for myself, I wouldn’t be competitive or anything like that.” (P3) O’Brien et al., 2021* | U |
|  | “It motivated me and drove my ambition to do more sports, respectively gymnastics, on a regular basis and the small daily successes supported that.” (participant, female, > 70 years, high level of education) (Wichmann et al., 2020) | U |
|  | “More individualization at the beginning would keep more people on board.” (participant, female, > 70 years, high level of education) (Wichmann et al., 2020) | U |
|  | “The exercise catalogue, which was divided into different segments, positively surprised me, respectively, enthused me moderately. Good, interesting exercises! Easy to do for everyone and efficient!“ (participant, male, high level of education) (Wichmann et al., 2020) | U |
|  | “Well, about the logging in regularly and taking notes every day, that was indeed a motivation. The stimulation to do a bit of something each day. I then made sure that I was always at the gym on Monday, Wednesday, and Friday, and on the other two days I tried to cycle a little or to jog once in a while, that is, something that I otherwise had so far avoided.” (participant, female, ≥70 years, high level of education) (Wichmann et al., 2020) | U |
|  | “Because it was fun to learn something about improving my physical and mental health in a group setting.” (participant, female, > 70 years, high level of education) (Wichmann et al., 2020) | U |
|  | “You know because I had only recently retired as well so it was thinking of some exercises that I could do or something I could do you know to get me out of the house rather than sitting reading and watching television all the while” (ID11 Male 70-74 years FAME) (Maula et al., 2019) | U |
|  | “We don’t mind doing these Pro-Age 65 exercises, as I say we do them every morning without fail, we always find time for that, then we start to get ready to go out or do a days work whatever” (ID26 Female 75-79 years OTAGO) (Maula et al., 2019) | U |
|  | “you get in a routine really and we have such a jolly time that… and I think we all felt that we benefitted hugely, that no it was good” (ID7 Female 70-74 years FAME) (Maula et al., 2019) | U |
|  | “I just carried on doing them because they were asking you how many times you had done them, if one a regular basis with your legs and the weights and all that sort of thing so I just did it and never thinking that this would be coming along now so I did them because they were part of the exercise” (ID20 Female 75–79 OTAGO) (Maula et al., 2019) | U |
|  | “I would have liked to have carried on doing it because filling those papers in as well every month gave you the incentive to do it” (ID30 Female 75-79 years OTAGO) (Maula et al., 2019) | U |
|  | “you have got to give them a goal and you have got to let them see that they have achieved something” (ID24 Male 75-79 years FAME) (Maula et al., 2019) | U |
|  | “I am used to having… doing things, physically, and on top of that exercising regularly in as an individual…………….. so I am quite capable of stringing together a programme that would suit me” (ID21 Male 70-74 years OTAGO) (Maula et al., 2019) | U |
|  | *“Imagine that I came home one evening and when I sat down in front of the TV, sitting down there is something most people do anyway, and the first thing happening is that the screen asks me How am I today? What do I need to do to feel better?” (Ehn et al., 2019)* | U |
|  | “But it is all the short distances you walk, if you summarize them every day. But that really adds up to something. Then you are adding on to the sum. That sounded a bit exciting”. (Ehn et al., 2019) | U |
|  | “It might come as a shock, to see the amount of sedentary behavior this time of the year”. (Ehn et al., 2019) | U |
|  | “But then you become motivated to do better in the next training session. I want to get thumbs up again.” (Ehn et al., 2019) | U |
|  | “My experience is that seniors used to say that they want to feel that they own the data themselves. That they want to have control of the information.” (Ehn et al., 2019) | U |
|  | *“If we talk about a target group that needs rehabilitation in order to be activated, then it can be a real failure if you have a really bad day and are not able to do anything. In that situation, to get digital feedback saying today you have accomplished nothing. When instead I could already, from the start, lower my goal. Because then I might feel cheered up despite the bad day with low energy. Because I did something. And I think that is important.” (Ehn et al., 2019)* | U |
|  | “I hope that by using my fitbit, I will walk more and win” (2A) (Abouzahra and Ghasemaghaei 2020) | U |
|  | *“*You young people think we cannot handle technology. You forgot that our generation invented computers” (7A)*. (Abouzahra and Ghasemaghaei 2020)* | U |
| Category 3. Social support and virtual interaction | *“I wouldn’t mind playing my grandson. [It] Would be a challenge, if I wasn’t successful that was fine, but this is something to do together.” (Hosseini et al. 2024, P7)* | U |
|  | *“I find Zoom fine. I mean, I don’t do as much of it as some people do. So I don’t have that wealth of background but I find it certainly works as far as having a face to face communication and direct interaction.” (older adult, male, 65 years) (Akinrolie et al., 2024)* | U |
|  | *“Participant I often participates in various events and posts . . . It seems that he has a sore throat, but he often takes various pictures and posts them, so I understand the pictures easily, even without his voice. It is easy to understand what he is doing.”— Participant A (Shinokawa et al. 2023)* | U |
|  | “If it could be set up for two players and one hit one colour*,*and one hit the other colour*,*that would be a challenge”*(P10). (Hosseini et al., 2024)* | U |
|  | "If we have a volunteer*,*hopefully*,*we can*.*Somebody that could be available"*(P5). (Hosseini et al., 2024)* | U |
|  | "You escorted me through… if I had been there by myself, I might have had difficulties" (P4) (Hosseini et al., 2024) | U |
|  | "If you weren’t here, I don’t know how I would go through" (P5) (Hosseini et al., 2024) | U |
|  | *“Well, I loved getting to know them [the exercise staff]. Where they lived. What they do. Whether they had a dog and their families and to visit socially. I think it was just as important socially for me as physically. I really, I enjoyed every one of the very well trained [exercise staff]” (F, 79) (Dunston et al., 2024)* | U |
|  | *“Yeah, and then I had a husband that was very supportive. You know he would exercise and then I felt like I had to exercise too.” (F, 74) (Dunston et al., 2024)* | U |
|  | *“The other thing I really like is they can watch me do the exercises and tweak the way I was doing them. If I was doing them a little bit off or not quite right I noticed there was a big difference when I was doing them correctly as far as how it would impact the results of the exercise.” (M, 69) (Dunston et al., 2024)* | U |
|  | “And the safety of myself because, you know, a lot of people don’t think that there’s COVID out there. Still COVID (is) out there” (older adult, female, 65 years) (Akinrolie et al., 2024) | U |
|  | “Counsellor really did remember from session to session, something would be said in one session, and counsellor would bring it up again. You know, in another session, that was pretty impressive. So, counsellor was obviously listening to me” (Older adult, female, 77 years) (Akinrolie et al., 2024) | U |
|  | “So, I was happy about that. I find older adults to be a lot easier to talk to, they’re a lot more comfortable and confident in themselves. So, I think that is something that just makes the conversations flow pretty easily” (counsellor 2) (Akinrolie et al., 2024) | U |
|  | “I think MI skills can definitely be applied virtually; I didn’t have any problems using any of the techniques. It’s all in the communication and communicating over Zoom was very easy” (counsellor 1) (Akinrolie et al., 2024) | U |
|  | “I think it sort of comes back to that idea of we’re both in environments where we’re comfortable. And that can do a lot for building rapport. So, if a participant [older adult] is in a space where they feel safe and comfortable, then they might feel more willing to open up to their counsellor and talk to them about any struggles that they’re facing” (counsellor 1) (Akinrolie et al., 2024) | U |
|  | “. . .like acceptance and compassion are not something that has to change for an online context, right? If you are here to try to legitimately help people and you care about them and want to listen to them, like you tried to be empathetic, so whatever barriers they might have or what their history might be. So, I think that wasn’t super hard to apply” (counsellor 2) (Akinrolie et al., 2024) | U |
|  | *“I just gave my sister one [WAM] for her birthday. And now, she calls me every day to tell me how many steps she’s done.” –Female, ever WAM user, “less active.” (Zytnick et al., 2023)* | U |
|  | *“I was not familiar [with WAM]…but [now that I am participating in this focus group] I know a little bit more.” –Male, never WAM user, “more active.” (Zytnick et al., 2023)* | U |
|  | *“ ::: (name) is just a good facilitator; he is fabulous:::” (p45/F/online) (Weselman et al., 2023)* | U |
|  | *“I feel the staff are very supportive and very friendly ::: they make everyone feel welcome” (p4/F/in person) (Weselman et al., 2023)* | U |
|  | *‘The pole walking is clearly a crowd favorite, everyone loved getting outside and the mood was really positive’ (researcher diary, 4 September 2021). (Weselman et al., 2023)* | U |
|  | *“ ::: I think it’s good for people who don’t have a big social circle or who don’t have many family members” (p45/F/online) (Weselman et al., 2023)* | U |
|  | *“The good thing is you get to connect with the same people in all the classes” (p36/F/online) (Weselman et al., 2023)* | U |
|  | *“I joined the community hub specifically to keep myself socially engaged, because I know this is a really important part of maintaining your health and well-being” (p47/F/online). (Weselman et al., 2023)* | U |
|  | *“I feel more a part of the community now” (p18/M/in person) (Weselman et al., 2023)* | U |
|  | *“I think that whole community activities were very good and drew a lot of more people into it” (p30/M/in person). (Weselman et al., 2023)* | U |
|  | *“there are a lot of women, that’s always good, a couple of the women were quite mature, and that’s inspiring” (p8/F/in person). (Weselman et al., 2023)* | U |
|  | *One participant had a more positive outlook, explaining that she worked harder to maintain connections during lockdown, she stated, ‘I think during COVID-19 lockdown, I forged stronger connections with the people’ (p8/F/in person). (Weselman et al., 2023)* | C |
|  | *Participant I frequently attends various events and posts about them… It seems that he has a sore throat, but he often takes various pictures and posts them, so I understand the pictures easily, even without his voice. It is easy to understand what he is doing. (Participant A) (Shinokawa et al., 2023)* | U |
|  | *“I am inspired by other people’s posts. Even if it is the same scenery or photo, I feel that there are different ways of feeling and seeing.” (Participant F) (Shinokawa et al., 2023)* | U |
|  | *“I think a post like that (where we can hear their wife’s voice in the background) is fine. I think it’s great that it conveys a sense of life.” (Participant C) (Shinokawa et al., 2023)* | U |
|  | *“I can see that other people are absent on Saturdays and Sundays, and on the contrary, there are people who are active on Saturdays and Sundays. I wonder what kind of person he is. It may be easier to meet and talk because I understand that.” (Participant I) (Shinokawa et al., 2023)* | U |
|  | *“The other day, I happened to sit next to one of the members at a community gathering New Year’s party. It was then that he realized for the first time that she belonged to the same group.” (Participant G) (Shinokawa et al., 2023)* | U |
|  | *“I want to cherish the long-awaited meeting because we met and worked together by chance.” (Participant F) (Shinokawa et al., 2023)* | U |
|  | *“After listening to the post, I thought that I would like to cooperate with everyone who has the same idea to liven up the meeting.” (Participant G) (Shinokawa et al., 2023)* | U |
|  | *“Even if I cannot meet or go out because of the new coronavirus infection, it is fun to connect with the members by using the communication app.” (Participant E) (Shinokawa et al., 2023)* | U |
|  | *“I think we can share our feelings. (I think it’s better to post what I think, even if I stumble or use short sentence.” (Participant C) (Shinokawa et al., 2023)* | U |
|  | *“The number of steps participant H takes every day is amazing. How does he do it?” (Participant B) (Shinokawa et al., 2023)* | U |
|  | *“I want to publicize what I have done so far, such as what kind of conversation is necessary when interacting with people, how I have maintained my health, etc.” (Participant G) (Shinokawa et al., 2023)* | U |
|  | *“Some participants enrolled through word-of-mouth, likening their participation to being in their “own little sorority or fraternity because we were talking about Bandit.” (Crane et al., 2023)* | C |
|  | *“I told all my family what I was doing… they would ask me each week how I did. They were very interested in my being there and my accomplishments.” (Crane et al., 2023)* | U |
|  | *“I liked having somebody in the room, because… if they walked out, we're there alone… They showed that they actually had an interest…they were very encouraging, they stopped if you were tired at all. You could stop at any time. They talked to you. And I thought they made the program work.”  (Crane et al., 2023)* | U |
|  | *“I would use that device. …I really like this because she’s always asking.., 'Are you ready?' So, you always have to give a response. You can’t just say oh well, I’d like to quickly go and get the bin out or what. You just can’t.” (Participant 3, female, age 67). (Jansons et al., 2022)* | U |
|  | *“(I enjoyed) that someone was talking to me when you live on your own.” (Participant 8, female, age 67). (Jansons et al., 2022)* | U |
|  | “It’s like when I went to the gym, and I have an instructor there, and he said, “Why do you want to be here? What exercises you want to do? Do you want to lose weight? Or you want to just have more agility? Or you want to have fun or whatever it is.” So it can be – then he went and selected five different programs and showed me how to do it. And then every second day I go myself and I do them myself. Some for the leg and the calf, others for the ankles, others for whatever.” (Participant 1, male, age 70). (Jansons et al., 2022) | U |
|  | “I’m really an emailer. And, you know, phone and text. Not a lot else in a way, really.” – P7, Female, age 70 (Simmich et al., 2021) | U |
|  | “I don’t like social media. I got on it in 2011, when we had the last major lot of floods in Brisbane. […] I really don’t like it for me, but I use it as a way of just staying in touch with, you know, what the boys are doing and some other people that I know. But I don't make a posting on it myself, ever.” – P6, male, age 68 (Simmich et al., 2021) | U |
|  | “My missus set up a Facebook page for me, but I’ve never used it. […] I’ve been thinking I should get in and redo it, because…my family…we don’t sort of stay in touch very well. Apparently I miss out on a lot by not having Facebook. Because they post all this stuff on Facebook, so I'm thinking I should get on Facebook.” – P10, male, age 62 (Simmich et al., 2021)" | U |
|  | “Somebody looking over your shoulder, who probably knows a damn sight more about it than what you do. To say ‘Well, hey you’re not doing anywhere near enough’, or, you know, ‘You’re doing well’, or ‘You’re overdoing it’.” – P10, male, age 62 (Simmich et al., 2021) | U |
|  | “I guess if it was your therapist or your doctor or whatever, you know, that’s more data for them on which to be able to shape whatever advice it is that they going to give you. So I can’t see anything other than a benefit in that.” – P6, male, age 68 (Simmich et al., 2021) | U |
|  | “I play a lot of games with my grandchildren […] We play Beggar My Neighbour, and Uno, […] and I really enjoy that. We love playing games together.” – P11, female, age 74 (Simmich et al., 2021) | U |
|  | “Sometimes they can be really fun. When we have the family over […],we have a Wii game […] You can have a lot of fun doing things like that. And that’s all movement and exercise too.” – P14, female, age 66 (Simmich et al., 2021) | U |
|  | “Without the interaction of the kids, to me, that sort of game…eh, does nothing for me. But with the interaction of the kids, I enjoy it.” – P5, male, age 70 (Simmich et al., 2021) | U |
|  | “Yeah, the granddaughters have a sleepover, we play Uno. And I teach them the oldies. And we have another game we play. But see, they’re all grown up now, so you don’t.” – P16, female, age 73 (Simmich et al., 2021) | U |
|  | “Some of the games you can’t play on your own, you need somebody to play with.” – P13, female, age 71 (Simmich et al., 2021) | U |
|  | *“So in the normal situation, I think, it, it would be quite interesting to be with a group because you’d be discussing your activity and you’d get ideas and tips from people, you know? And so, I think it, it would be very interesting to use as part of a group, but it was certainly very interesting to use it on an individual basis as well.” (P11) (O’Brien et al., 2021)* | U |
|  | “The group helps me to stick to the program.” (participant, female, ≥70 years, medium level of education) (Wichmann et al., 2020) | U |
|  | “Yes, it would be nice if it were a mixed group and not only women would be there.” (participant, female, > 70 years, medium level of education) (Wichmann et al., 2020) | U |
|  | “Yes, I find it good, if my friend with whom I already do a few things would also take part.” (non-participant, female, ≥70 years, medium level of education) (Wichmann et al., 2020) | U |
|  | “Well, a professional qualification like the uh, there in, in the fitness field. That people are also trained, not only as fun organizers, but have medical knowledge. That I would like that, yes.” (participant, female, ≥70 years, high level of education) (Wichmann et al., 2020) | U |
|  | “They were approachable, we could ask them and they were forthcoming. I found that to be very good. They were able to convey it very well and I found that to be very, very good.” (participant, female, > 70 years, medium level of education) (Wichmann et al., 2020) | U |
|  | “Unfortunately, it was like this. In the beginning, when the program started, we were about 20 people, but only four or five showed up for the meetings. These were then also almost always the same people. The one or the other then also came along, but otherwise there were mainly five or six people. Too few. And I really think the exchange is important, so that one can also hear from the others how they go about it (laughs).” (participant, male, ≥70 years, high level of education) (Wichmann et al., 2020) | U |
|  | “Representatives from sports club should present their activities during group meetings.” (participant, male, < 70 years, high level of education) (Wichmann et al., 2020) | U |
|  | “Well, the chemistry just has to be right. It somehow has to be people, that one somehow has some common interests.”(participant, male, < 70 years, medium level of education) (Wichmann et al., 2020) | U |
|  | “Well, in order not to have large differences (laughs), it would be nice if the same age groups would be there, I’d say it should be from 60.” (participant, male, ≥70 years, high level of education) (Wichmann et al., 2020) | U |
|  | “But somewhere where one says, okay, they accept you, I accept them. But let’s say like 35 to 40 year olds.” (non-participant, male, ≥70 years, medium level of education) (Wichmann et al., 2020) | U |
|  | “This is my personal …, as I said, I met two ladies who I have contact with because I had been looking for company and we will deepen this relationship.” (participant, female, > 70 years, medium level of education) (Wichmann et al., 2020) | U |
|  | “Yes the social side of it yes because we did… You know when you’re retired and not going to work, it is trying to find something else and still meeting people” (ID1 Male 70-74 years FAME) (Maula et al., 2019) | U |
|  | “It was actually one of the people that went to the Pro-Active……they started going to Tai Chi and it was him that told me about this Tai Chi classes so we enrolled in that, we both enrolled on that so it was you know it was somebody we had met at those classes that talked us in to going or told us about this Tai Chi so we thought we would give it a go and erm like I say because we were all beginners” (ID11 Male 70-74 years FAME) (Maula et al., 2019) | U |
|  | “Yes because we’re both more or less go to the same type of things and one pushes the other one on shall we say” (ID27 Male 75-79 years OTAGO) (Maula et al., 2019) | U |
|  | “Yes because we work together, if we’re doing anything it is usually done together” (ID26 Female 75-79 years OTAGO) (Maula et al., 2019) | U |
|  | “And I was showing him some of the exercises and it is when hearing him say that would do you good, now that would be good for you...” (ID2 Female 80-84 years OTAGO) (Maula et al., 2019) | U |
|  | “We used to say that, for example, when we go bowling, that half of the amusement is the bowling and the other half is to get out and socialize with others. That is almost more important. For most of us it is at least more important than getting a good result”. (Ehn et al., 2019) | U |
|  | *“If we [in the associations] had anything enabling us to attract for example 10–12 persons on our group walks instead of the seven persons that usually come, then we would have made an impact for those who really need the walks.” (Ehn et al., 2019)* | U |
| Category 4. Environmental factors | *“Yeah, for me the fact that [it] is offered [by] distance, telemedicine, is the only reason I can do it ‘cause I can’t make the 6-hour round trip drive to go in-person.” (P69) (Dunston et al., 2024)* | U |
|  | *“Financially the amount one had to pay allowed me to be in this one-on-one exercise program with professionals that can help.” (F, 74) (Dunston et al., 2024)* | U |
|  | “And so they will be at home, this is where they’re trying to get more active, and maybe taking that meeting at home, they’ll be a little bit more aware of the real-life barriers that have been coming up with them in terms of their physical activity” (counsellor 1) (Akinrolie et al., 2024) | U |
|  | “So, I was happy about that. I find older adults to be a lot easier to talk to, they’re a lot more comfortable and confident in themselves. So, I think that is something that just makes the conversations flow pretty easily” (counsellor 2) (Akinrolie et al., 2024) | U |
|  | *“ ::: a great opportunity to try different things at minimal or zero cost” (p47/M/online) (Weselman et al., 2023)* | U |
|  | *“::: whole area felt quite inclusive” (p7/F/in person). (Weselman et al., 2023)* | U |
|  | “Yeah. I went away to Byron Bay and I only took carry on. I squashed it in there, so it was all right.” (Participant 14, male, age 74). (Jansons et al., 2022) | U |
|  | *“I find, I suppose as a matter of interest in the current lockdown, ehm and I’m in the vulnerable section ‘cause I’ve had [an illness] in the last 5 years. So, ahm, I find it’s very useful to keep motivated to do a bit of ehm you know, jogging on the spot or whatever because I’m indoors now for two weeks. So its particularly useful, yeah.” (P8). (O’Brien et al., 2021)* | U |
|  | *“Of course, there is a financial span. It should not be too expensive, but a certain fee, what one also pays for a sports club membership or wherever, no problem at all.”(participant, female, < 70 years, medium level of education) (Wichmann et al., 2020)* | U |
|  | *“Well, it should be somewhere close, okay? I wouldn’t want to like have to drive far to get there, that I first have to drive 20 km or so.” (non-participant, male, ≥70 years, high level of education) (Wichmann et al., 2020)* | U |
|  | *“The exercises can be mastered by experienced and by untrained people of that age group. Little time is required.” (participant, male, ≥70 years, high level of education) (Wichmann et al., 2020)* | U |
|  | *“I was at the home, doing the home, I would have rather have gone to the classes because I think it encourages you more, when you are at home you have got no stimulation more or less when you’re at home but when you are in a class you try and do the same as everybody else don’t you?'” (Maula et al., 2019)* | U |
|  | *“You don’t always want to be going down town” (ID2 Female 80-84 years OTAGO) (Maula et al., 2019)* | U |
|  | *“it does make you go because you have paid for it in advance” (ID11 Male 70-74 years FAME) (Maula et al., 2019)* | U |
|  | *“I mean when you have got a pension you do struggle to pay for such as gyms and things like that so if I could find something that was reasonable then I would probably do more” (ID30 Female 75-79 years OTAGO) (Maula et al., 2019)* | U |
|  | *“yes you need transport, which I have got……… I have got a free bus pass and the buses are only there across the road” (ID28 Female 75-79 years OTAGO) (Maula et al., 2019)* | U |
|  | *“Accessible Transport ‘yes and physically I wasn’t driving then so I couldn’t get to where they were” (ID10 Female 85-89 years FAME) (Maula et al., 2019)* | U |
|  | *“Erm… the weather, you know like I was saying if it is not a very nice night you obviously don’t go or if it is cold or snow” (ID1 Female 70-74 years OTAGO) (Maula et al., 2019)* | U |
|  | *“Well you read the media of people being attacked, today there is someone being attacked and phone taken off her, that sort of thing” (ID29 Female 70-74 years OTAGO) (Maula et al., 2019)* | U |
|  | *“As long as it is sort of day time and you’re not travelling too far” (ID6 Female 70-74 years FAME) (Maula et al., 2019)* | U |
|  | *“I got fitbits to my grandchildren in Christmas, they loved them and asked me why I don't use one” (1A) (Abouzahra and Ghasemaghaei 2020)* | U |
|  | *“Many of my friends use fitbit or similar wearables. They said fitbit was very good in tracking their steps and calories” (10A). (Abouzahra and Ghasemaghaei 2020)* | U |
| **Synthesised findings 2. Structural and personal limitations in digital health engagement** | ***Illustrative Quotes*** | **Credibility** |
| Category 1. Technical barriers and access difficulties | *“I think it’s, the technology is intimidating to me, like, is it hard to set up, or how to use it… Just, is it going to be challenging to set it up, not use it, but to set it up?” (Zytnick et al., 2023; P female)* | U |
|  | *“I discovered that I was folding laundry and I was reaching my goals because it’s reading your arm rather than your leg. So it’s not accurately doing steps.” (P7) (Li et al., 2024)* | U |
|  | *“Only at the very end—the last two days—I had discovered that the plastic film was still on the back of the watch, so it didn’t have contact with my skin” (Study 1 Participant #2). (Li et al., 2024)* | U |
|  | "Music is a noise" (P11). (Hosseini et al., 2024) | C |
|  | “I was happy to see how easy it was, except about the controllers. I never got master of those.” (P7) (Hosseini et al. 2024) | U |
|  | *Some participants found the headset weight burdensome, further affecting their experience and interaction with the game. (Hosseini et al., 2024)* | C |
|  | *“It took a while. I had to figure out how exactly to set up my ipad. Then move it when I went from floor to standing and that, but we’ve got it figured out now. But, it took a while.” (F, 72) (Dunston et al., 2024)* | U |
|  | *“Well, my wife and I both use one and go on walks together, and we have completely different readings. She seems to walk a hell of a lot more than me.” –Male, ever WAM user, “less active.” (Zytnick et al., 2023)* | U |
|  | *“I think that having to wear [WAM], remember to put it on every day, and the wrist-worn ones you have to charge every -- I’m charging an iPhone, I’m charging an iPad, I’m charging a computer. I’m tired of charging. I don’t know, I think it’s inconvenient.” –Female, never WAM user, “less active.” (Zytnick et al., 2023)* | U |
|  | *“The graphics I thought were very crude. I think they could have been more pixels in the display to make the pictures easier to understand.” [Participant 5] (Stawarz et al., 2023)* | U |
|  | *“That equipment was quite awkward, you know, the cables and the fittings and the plugs didn’t seem to fit very securely. It was all kind of it all looked a bit fragile.” [Participant 3] (Stawarz et al., 2023)* | U |
|  | *“The technology is too crude and intrusive at this early stage, compared with either a) doing without or b) doing something clever with it.” (Stawarz et al., 2023)* | U |
|  | *“I think [the prototypes] just feel, um, they don’t feel user friendly and they don’t feel...they feel like old technology. I think it would need to have a screen; it would need to look like a phone; it would need to have a digital reader, you know, all of that, like the apps we have on our phone.” (P3) (Stawarz et al., 2023)* | U |
|  | *“They just needed to be there to advance the game when you finish a level so you can change to a new level.” (Crane et al., 2023)* | U |
|  | *“The voice interaction is very problematic. It does not understand a clearly enunciated yes or no, and I think there’s a reason for that. But you’ll see lots and lots of examples where it just refuses to understand a simple yes or no, or any other command, and given that it’s voice-driven that’s a critical failure.” (Participant 6, male, age 77). (Jansons et al., 2022)* | U |
|  | *“No, the main negative was it may be my (Internet WiFI) connectivity here, I notice on Saturdays it always goes bad for the last three or four weeks, when people have been escaping from Melbourne and bringing their devices down, swamping the bandwidth. Last Saturday it took one session when I waited for it, a good three quarters of an hour. So the “exercise program” doesn't work when your connectivity is not good.” (Participant 11, male, age 71). (Jansons et al., 2022)* | U |
|  | “Yeah, even if it was saying ‘Too fast. Too fast,’ because that’s what I tend to do, everything too fast. Or you know, it might say ‘Just one more,’ you know, that sort of thing. Yeah, I think that sort of feedback would make it even more engaging for people” (Participant 9, female, age 65). (Jansons et al., 2022) | U |
|  | “You don’t get feedback, so for somebody who’s not maybe that good at doing their exercises, I don’t know how you would be able to but it would be great if they would get a bit of a feedback on it, whether there’s a physical way you could get somebody to do the exercise on a certain spot and put the camera on a certain spot and then it would be able to measure – or what, I don’t know. Maybe a future version.” (Participant 9, female, age 65). (Jansons et al., 2022) | U |
|  | *“Well I had the camera turned off, so I’m not sure – and because I live alone, there’s not too many conversations going on around a machine, but if you had a private conversation you probably wouldn’t have it in front of Alexa. But I don’t know. If you haven’t turned her on I don’t know if there’s any recording happening anyway. I don’t know. I don’t know of that privacy.” (Participant 12, female age 68). (Jansons et al., 2022)* | U |
|  | *“I have turned off the camera except talking to you now. Yes, I suppose with the camera on I would have issues with that, in general, not from Deakin University, but just from Amazon up there in the Cloud, and who else is on it and so on.” (Participant 15, female, age 74). (Jansons et al., 2022)* | U |
|  | *“It did concern me, because you don’t want to think that you’re being listened to all the time and watched all the time, however it was alright once I found where to turn off the camera.” (Participant 7, female, age 67). (Jansons et al., 2022)* | U |
|  | *“Well, I’m a little bit—I mean in terms of the exercise and stuff not really, but if I had to—like, if it was a different kind of program where it’s involving a lot of private things like medical stuff or opinions about things or whatever I might, yeah, think twice about that because the stuff that it’s Alexa or a Google Home thing where they do—you don’t know where your information is and all that sort of thing. Yeah, I mean I’m a bit, not suspicious, but I’m wary of the devices just generally, but in terms of just data for the exercise it’s not an issue for me. It’s only if it’s asking personal information.” (Participant 13, female, age 66). (Jansons et al., 2022)* | U |
|  | *“Well, I wouldn’t leave it on in between sessions and I wouldn't leave the video on at all.” “It's sort of really amazing when you just have a passing comment to someone during the day, your phone picks it up and then there's an ad about it two hours later.” (Participant 11, male, age 71). (Jansons et al., 2022)* | U |
|  | “I remember sitting in front of a computer when we first got it and I thought, how do I turn it on? Because nobody told me. So I can remember I got the manual out and I found out how to switch this on. And they were the old computers. And I think those days…technology in computers is so advanced now. That I’ve forgotten. Remember have to put in discs and I don't know. Too hard.” – P2, female, age 76 (Simmich et al., 2021) | U |
|  | “You might walk for 40 minutes but you can actually do nothing in that 40 minutes. And unless you measure it with a pedometer that puts a number basically on what you're doing.” – P10, male, age 62 (Simmich et al., 2021) | U |
|  | “You just flip it on and go for a walk. And you think, ‘Oh, is that all the steps I’ve done? Feel like I’ve done a thousand.” – P16, female, age 73 (Simmich et al., 2021) | U |
|  | “No, we don’t have an Xbox, we don't have any of this machinery stuff. The grandkids have that. I’ll have to ask them.” – P13, female, age 71 (Simmich et al., 2021) | U |
|  | “We haven’t got a game and I wouldn’t go out and buy [a Nintendo] Wii to try it out.” – P2, female, age 7 (Simmich et al., 2021) | U |
|  | “If there is something, some instruction with movement, to assist my breathing, yes, I would be open to a program like that. It would have to be clear. It would have to not have too much periphery that is unnecessary. It doesn’t have to try to motivate, to entertain me as such, because it's not for entertainment” – P7, female, age 70 (Simmich et al., 2021) | U |
|  | “You want to be able to see if you’re improving. You want it to say if the exercise to improve the number of times you do something improves your ability to lift a weight. I’d like it to be measurable. If it was a game, it wouldn’t matter. But if it was an ‘exercise game’, I’d like it to be measurable.” – P18, male, age 59 (Simmich et al., 2021) | U |
|  | “It’s also a case of … ‘Hmm, why are we doing this?’ in a way. There could be that question, because you’re just standing there and it’s all make-believe.” – P1, female, age 66 (Simmich et al., 2021) | U |
|  | “[Nintendo] Wii, is like, it’s a simulation, yeah? I think I’d rather go outside and play on a tennis court. I don’t know, I haven’t played it. But I don’t think it would have the same impact as you would out and hearing a ball hit a tennis racket. You can go out into a field and do that exercise and it’s free. And it’s fresh air.” – P2, female, age 76 (Simmich et al., 2021) | U |
|  | *“I didn’t really understand how to use it [the device] properly, I’d be able to count the steps and then the steps would […] be gone, and there was days I, I didn’t know how many steps I had, I was only judging it using an average.” (P4) (O’Brien et al., 2021)* | U |
|  | *“What I did find about the exercise was it only records steps, you know. I was, we say now in aqua aerobics, I would be doing a lot of movement in that but you’re not recording anything in that kind of exercise, you know? It didn’t really sort of ehm, describe how, how much exercise I was doing.” (P4) (O’Brien et al., 2021)* | U |
|  | *“No, I didn’t [compare steps] because we seemed to go in kind of individually, do you know what I mean?” (P3) (O’Brien et al., 2021)* | U |
|  | “Perhaps a CD with the exercises, which one can look at later on and check if the motions are still correct. It can also be for a price.” (participant, male, < 70 years, high level of education) (Wichmann et al., 2020) | U |
|  | “Entering something into the PC every day was too much.” (participant, female, < 70 years, high level of education) (Wichmann et al., 2020) | U |
|  | “The synchronization with the Fitbit-App was at times very tedious (several attempts required).” (participant, male, < 70 years, high level of education) (Wichmann et al., 2020) | U |
|  | “To design the website better: i.e. make it easier for senior participants to understand.” (participant, female, ≥70 years, medium level of education) (Wichmann et al., 2020) | U |
|  | “There were participants who had major problems installing the program. Their questions were not, respectively, could not be answered, they gave up, they were somehow excluded and I never saw them again.” (participant, male, > 70 years) (Wichmann et al., 2020) | U |
|  | “(…) it would therefore have been better to focus on the title “fit” and to place the sports part at the beginning. The questions and explanations regarding the technique can then be explained at the end if needed.” (participant, male, < 70 years, high level of education) (Wichmann et al., 2020) | U |
|  | “Not all participants have access to the internet, particularly older participants. Hard copies would have been better.” (participant, female, < 70 years, medium level of education) (Wichmann et al., 2020) | U |
|  | “That (a step counter) was not so fun. You needed to remember to put it on.” (Ehn et al., 2019) | U |
|  | “I think many people are afraid of technology. These things with credit cards and phones and people calling pretending to be…That strikes back on all technology in a way.” (Ehn et al., 2019) | U |
|  | *“At many times, fitbit would not wakeup and I couldn't see my data. At other times, it did not show any heart rate. This made me so frustrated” (7B). (Abouzahra and Ghasemaghaei 2020)* | U |
|  | *“Sometimes while working out, I could feel my heart beat was high but the fitbit did not show that” (1B) (Abouzahra and Ghasemaghaei 2020)* | U |
|  | *They handed us their phones to set up the wearable device software, and they were skeptical on whether they will be able to effectively use their wearable device. This attitude was clear in comments like*“You can't teach an old dog new tricks” (3A) or “I have never been good with technology” (6A). *(Abouzahra and Ghasemaghaei 2020)* | C |
| Category 2. Physical, psychological and social  problems | *“I was annoyed with myself that my left hand was not as accurate as my right hand. I’ve always been right-handed. I don’t use my left hand very much at all.” (P8) (Hosseini et al. 2024).* | U |
|  | *“When she arrived in one morning and I think she’d already done about five or six thousand steps, oh God, put me to shame, so I wouldn’t be able to compete with them like.” (P2) (O’Brien et al., 2021)* | U |
|  | *“. . .Even reading kind of like subtle facial expressions, or, like those kinds of nonverbal responses, and that type of thing, obviously, can’t quite be figured out in the same way as an in-person scenario.” (P2) (Akinrolie et al. 2024)* | U |
|  | "Perhaps for an older person, it must be slower" (P7). (Hosseini et al., 2024) | U |
|  | “Like for the participant (older adults) I already mentioned, I had a lot of trouble being able to get a turn to speak. And I think a lot of that has to do with the fact that it was on Zoom” (counsellor 2) (Akinrolie et al., 2024) | U |
|  | “. . .. there are a lot of nonverbal cues that can be picked up, of course, much better in a physical meeting, you know, than here [virtual], I mean, we’re dependent upon reading everything from our. . ., basically for the top of our head to the shoulders. . .” (older adult 2, female, 69 years) (Akinrolie et al., 2024) | U |
|  | *“I just get out and exercise, without worrying about cleaning the dusty house” (p4/F/in person) (Weselman et al., 2023)* | U |
|  | *“I wasn’t terribly good with exercises because of rheumatoid arthritis” (p41/M/online). (Weselman et al., 2023)* | U |
|  | *“I did find that I had some memory issues towards the end of the test and a few days I would just forget to put it on” (Study 2 Participant 4) (Li et al., 2024)* | U |
|  | *“The balance exercise is quite easy, and the sit-to-stand is more strenuous. It’s hard work. It takes more energy and makes me tired.” (P5) (Stawarz et al., 2023)* | U |
|  | *“It would be nice to have a wider range of exercises. I mean, as you get old your backs get stiff. You get stiffness in lots of joints. I think that it could be done to use more joints as a body, try and create more flexibility.” (P5) (Stawarz et al., 2023)* | U |
|  | *“I have really bad knees that I couldn't stand for long periods of time, but the researchers allowed you to rest.”  (Crane et al., 2023)* | U |
|  | “I used to do some of these exercises with the exercise physiologist, I know how you’re meant to do them. But for some people, they probably need a little bit more guidance on some of the exercises. Like I know you do say ‘hold your bum in’ or whatever, but some of them it’s quite important that they do it correctly. So it’s tricky because the first time they do it they need the full instruction and then after that you’re just say reminding them what the exercise is, so.” (Participant 9, female, age 65). (Jansons et al., 2022) | U |
|  | “When the grandkids were here they were doing some exercises…some exercise things on the…on the TV and I thought, ‘Oh, don't think I could be doing that’. […] I have to be very careful what I’m doing because of my osteoporosis. Don’t want to start breaking bones otherwise I'm really badly off.” – P13, female, age 71 (Simmich et al., 2021) | U |
|  | “Yes, it was a bit too long I have to say, for me that is. One somehow loses interest a bit afterwards, well, it’s the same thing over and over again. In the end it is only then. At some point one just says” Oh well, okay.” (participant, female, < 70 years, medium level of education) (Wichmann et al., 2020) | U |
|  | “I think once you stop exercising you stiffen up and that then takes a lot of coming back if you don’t keep yourself supple and mobile and well you become a cabbage” (ID26 Female 75-79 years OTAGO) (Maula et al., 2019) | U |
|  | “I was more flexible, I do have a bad back which I have had for erm along… decades” (ID4 Female 70-74 years FAME) (Maula et al., 2019) | U |
|  | “I can’t run anymore, the body is willing but the flesh is weak, the legs won’t go” (ID12 Female 80-84 years FAME) (Maula et al., 2019) | U |
|  | “I think one thing that has, I have thought about for the elderly and the reason why elderly people don’t do exercise is so many of them start going on medication for one thing and another and statins and erm a lot of medication does affect you and makes you feel lethargic and tired and so I think one of the main reasons with the elderly in stopping doing exercises it is tablets” (ID8 Female 75-79 years FAME) (Maula et al., 2019) | U |
|  | “I mean I have got arthritis all over the place, in my spine and in my neck and that sort of erm stops me doing anything too energetic” (ID13 Female 70-74 years FAME) (Maula et al., 2019) | U |
|  | “I am having a little problem with my knees so I don’t walk as much as I used to” (ID 23 Female 80–84 years OTAGO) (Maula et al., 2019) | U |
|  | “It is very difficult, you know people over 55, they’ve started to think they are getting old, and what do I want to do exercise for? Which I can’t… that is not in my mind but I can see this in people that I talk to you know of my age you know, what the hell do I want to do exercise for?” (ID5 Male 80-84 years FAME) (Maula et al., 2019) | U |
|  | “You wonder if it is through doing them that you have got these pains you know” (ID28 Female 75-79 years OTAGO) (Maula et al., 2019) | U |
|  | “Oh well I am well in my 70’s so you know I have a right to sit down and do nothing. I have got a friend like that and everything I do she says oh I bet you were tired weren’t you?” (ID8 Female 75-79 years FAME) (Maula et al., 2019) | U |
|  | “as you get older if you are on your own you need company so there is no company is there sat here? You know what I mean?” (ID28 Female 75-79 years OTAGO) (Maula et al., 2019) | U |
|  | “I can’t go any further and my legs just seize up and I feel mentally tired as well as physically tired” (ID30 Female 75-79 years OTAGO) (Maula et al., 2019) | U |
|  | “I think you can quite easily get depressed when you’re really tired so you have to sort of shake your feathers and say oh come on get on with it” (ID6 Female 70-74 years FAME) (Maula et al., 2019) | U |
|  | *“Among persons with weak balance that might have fallen. There is often, a not very much expressed, but a certain doubt to be active. And maybe above all else doubts, to be active by oneself.” (Ehn et al., 2019)* | U |
|  | *“I think that there is also a downside to this, a risk I can see is if the technology is used in order to replace any form of human contact. Especially when it comes to the oldest seniors with cognitive failure, then it is important to get another form of support, otherwise the technology will only become an obstacle.” (Ehn et al., 2019)* | U |
| Category 3. Individual and environmental limitations | *“I have a very small space, probably about 400 square feet. Not very big. For me it was really hard. They [the exercise staf] wanted to see me and I’m trying to exercise and I’m trying to hold my phone at the same time. That doesn’t work, because they want it at a certain level and want to be able to see you from head to toe while exercising.” (F66) (Dunston et al. 2024)* | U |
|  | *“So, you put out all that money and then you sort of use it in the beginning. But then I could see myself not using it, so then I’ve spent that money and I don’t use it. It just goes in my junk drawer.” (Zytnick et al. 2023)* | U |
|  | *Participant mentioned feeling warm during the gameplay and needing to turn on a fan, suggesting that the game provided a demanding PA that raised their body temperature. (Hosseini et al., 2024)* | C |
|  | “When I specifically asked for something and I don’t get a comment, the comment might be I’m sorry, that’s outside the boundaries of this study. But I didn’t really get an answer. It was just glossed over. . ..” (Older adult, male, 72 years) (Akinrolie et al., 2024) | U |
|  | *“I just want to say, a lot of us might want to do less. I am not interested in doing more physical activity than I’m already doing.” –Female, never WAM user, “more active.” (Zytnick et al., 2023)* | U |
|  | *“I wouldn’t really care to have [WAM] particularly. I mean, I do what [PA] I would do, and I try to do more. But I don’t feel that I need to categorize every darn thing I do.” –Female, never WAM user, “more active.” (Zytnick et al., 2023)* | U |
|  | *“I had no idea. The only exercise I do is when I come here [to the RSC].” –Female, never WAM user, “less active.” (Zytnick et al., 2023)* | U |
|  | *“I think that the people who greet you at the front door make or break the program, they had quite a domineering lady there that made me feel quite inadequate so I never went back” (p7/F/in person). (Weselman et al., 2023)* | U |
|  | *“You have got to regiment yourself to do these things haven’t you?” (ID29) (Maula et al., 2019)* | U |
|  | *“Yes I think the weather does, I think you know if it was pouring down with rain or something like that, I would probably be reluctant to go out and walk far in it.” (ID11) (Maula et al., 2019)* | U |
|  | *“I think gym costs are one of the biggest problems today” (p30/M/in person). (Weselman et al., 2023)* | U |
|  | *“::: unfortunately it was a little bit far for me to come regularly for activities” (p14/F/in person) (Weselman et al., 2023)* | U |
|  | *“I did it on Zoom and I could not say that this made me feel connected to the community ::: though it was strange as the camera was set-up to the side of the people, so they didn’t look straight [at the camera]” (p36/F/online) (Weselman et al., 2023)* | U |
|  | *“it tends to be more women, it would be better to have more men attend, so they could benefit from what the program offers” (p24/M/in person) (Weselman et al., 2023)* | U |
|  | *One participant commented that they would have preferred to be there in person for a better experience, as only a limited number of participants were allowed in the hub at one time. ‘There were noticeable restrictions ::: during the program’ p41/M/online) (Weselman et al., 2023)* | C |
|  | *“I’m not into where you have to discuss your feelings::: it’s not for me” (p4/F/in person). (Weselman et al., 2023)* | U |
|  | *“I’ve got lots of things all over the place. For this trial, I put them in the kitchen. But it would be in the way if I left there every day. I’d have to find somewhere else.” (P1) (Stawarz et al., 2023)* | U |
|  | *“If I kept it there during the day, I could easily trip on it. Anybody could trip or slip as well because I have a wooden floor so it could slip quite easily.” (P2) (Stawarz et al., 2023)* | U |
|  | *“I can take unusual pictures if I go to a strange place. Because I cannot go out owing to COVID-19, I do not have any pictures to post.” (Participant C) (Shinokawa et al., 2023)* | U |
|  | *“For now, because I cannot meet the members owing to COVID-19, I can only get in touch with them through this app, so I am still a little dissatisfied.” (Participant C) (Shinokawa et al., 2023)* | U |
|  | *“Yes, that’s convenient. Especially now that I’ve had the cataract operated on, it’s quite hard for me to read small print so I’ve got to find glasses which are never far away.” (Participant 15, female, age 74). (Jansons et al., 2022)* | U |
|  | “Oh yeah, I think probably too, coming into it at mid-70s almost, I have a problem remembering all the different processes. There’s…if I push this, that will happen, then I have to push that to get this. Whereas obviously as you would know my grandkids, the little ones, they know exactly what to push” – P3, female, age 76 (Simmich et al., 2021) | U |
|  | “I’m an age group when whole floors of the city building were given to a computer. […] Back then it was a case of ‘You don’t press enter until you make sure everything is right’. So I find it really hard how everyone, and even little kids, just go away and bash away everything, and you can pick it up. […] I was initially trained, you don’t go further until you make sure it's right. Well now every bit of technology I pick up, it doesn’t matter, you’ll be able to recover it, just keep going. So that's the big thing I’ve got to fight with it.” – P18, male, age 59 (Simmich et al., 2021) | U |
|  | “I just found the challenging annoying. I said ‘Stop challenging me because it’s just…it’s annoying me. I’m not enjoying my physical activity as much’. It certainly made me more active, I have to say. I have to admit that. Because I pushed myself to do more steps, but at the same time for me it took a bit of the enjoyment away. […] I wouldn’t keep it up, if somebody kept pushing me to do it, I just wouldn't keep that up.” – P14, female, age 66 (Simmich et al., 2021) | U |
|  | “I wouldn’t like to run in last. You know, in a competition, second-last, maybe third or fourth. But if I had to win it, I’d most certainly do my utmost to try.” – P8, male, age 59 (Simmich et al., 2021) | U |
|  | “I think it’s the whole competitive thing about games that does not particularly appeal to me. Because somebody’s got to win and somebody’s got to lose.” – P3, female, age 76 (Simmich et al., 2021) | U |
|  | “I’m just not into, you know, comparing myself to other people, because…like I know we’re all different, and I’m not interested much. […] I don’t want to be in constant competition, you know.” – P14, female, age 66 (Simmich et al., 2021) | U |
|  | *“Do you know what? It didn’t make a difference, really, it didn’t. I didn’t miss it when I finished with it, do you know that kind of way?” (P3) (O’Brien et al., 2021)* | U |
|  | *“I suppose I’m not sure I got really into it. Ahm, because I was fairly active anyway. So, it didn’t seem anything different.” (P5) (O’Brien et al., 2021)* | U |
|  | *“No, I didn’t [buy an activity tracker] and actually I was tempted, I was saying to myself do you know now with this lockdown and everything […] you can only go out once a day and the rest of it, I would have gone on longer walks, but now I can just do […] the two kilometres and what have you. So, I’m kind of more restricted and I would have found it more interesting to see how much movement I was doing, you know?” (P3) (O’Brien et al., 2021)* | U |
|  | *“Whereby it is not always good when there are couples in the group, they often argue.” (non-participant, female, ≥70 years, high level of education) (Wichmann et al., 2020)* | U |
|  | *“Perhaps participants should have been separated more according to their level of performance. Particularly for the strength training I would have liked to have other different exercises. The same exercises for ten weeks were monotonous and.” (participant, female, > 70 years, medium level of education) (Wichmann et al., 2020)* | U |
|  | *“I would have liked to have someone to assist me, at least when doing the first exercises, who would check whether the exercises were being done correctly.” (participant, female, < 70 years, medium level of education) (Wichmann et al., 2020)* | U |
|  | *“It was too much theory and too little exercising together.” (participant, female, < 70 years, medium level of education) (Wichmann et al., 2020)* | U |
|  | *“Well, there I would say if it has to be, then close to here in the area, but not too … if I first have to go to the other side of the town,, to the stadium or so in that direction, that is always quite a ride for us. Also not long, but not such that one is already tired from exercising. To the university, that would also be quite fast.” (non-participant, male, < 70 years, medium level of education) (Wichmann et al., 2020)* | U |
|  | *“When there’s a lot of traffic I would say I am there in 15 to 20 min. That would be possible, yes. One could also use the bike if need be, isn’t it? Well, but I wouldn’t really want to cycle into the city center or wherever else. I wouldn’t have/ I wouldn’t do that. That would be too far for me. That is a quarter of an hour, maximum 20 min.” (non-participant, female, > 70 years, medium level of education) (Wichmann et al., 2020)* | U |
|  | *“And I would even go as far as Osterholz, they are supposed to also have a nice swimming pool.” (non-participant, male, < 70 years, medium level of education) (Wichmann et al., 2020)* | U |
|  | *“And the venues, they were not appropriate. Well, I think it is probably difficult to find a suitable location. We sometimes had to do something in a very small area. I mean, it was nobody’s fault, in this, that was in the multigenerational house, sometimes we had the large room, then it was super, there one could move around.” (participant, female, > 70 years, medium level of education) (Wichmann et al., 2020)* | U |
|  | *“If one does sports, then one can’t only do so in a gym or in such places where there are appropriate (mats?). I think it was okay like that, it was a closed room.” (participant, male, ≥70 years, high level of education) (Wichmann et al., 2020)* | U |
|  | *“Yes, it should really be a location where there are other things nearby that one needs to do. Let’s say for example if one can do the shopping, other things, that is, everyday stuff things that one can do. So that I can say, okay, “I’ll do a bit of shopping, stop by the post office and then I have to do this and so forth.” That it’s in such an area. Well, and if one also had the chance to be inside or outside, depending on the time of the year, there would be nothing wrong with that, it doesn’t always have to be somewhere outside or only inside, but rather such that one would have different possibilities.” (participant, male, ≥70 years, medium level of education) (Wichmann et al., 2020)* | U |
|  | *“Well, it would have to be a gym where one, we at times were, okay, one could somehow change clothes. Those who came by car were already wearing their sports clothes.” (participant, female, < 70 years, medium level of education) (Wichmann et al., 2020)* | U |
|  | *“Larger rooms. I don’t like to move around if I’m touching strangers all the time and a good room atmosphere is important to me.” (participant, female, ≥70 years, high level of education) (Wichmann et al., 2020)* | U |
|  | *“It’s very complex and time consuming.” (participant, female, < 70 years, medium level of education) (Wichmann et al., 2020)* | U |
|  | *“Uh, and it has to take place at least twice a week. It can also be even three times, but I think twice a week is very important to be able to keep in tune. Plus, there is also the possibility to do the exercises at home but I am a little phlegmatic I.” (non-participant, female, ≥70 years, medium level of education) (Wichmann et al., 2020)* | U |
|  | *“In principle, I’m a bit reserved when it comes to organized activities. I mean, groups, I am not a big group person.” (non-participant, male, ≥70 years, high level of education) (Wichmann et al., 2020)* | U |
|  | *“I wasn’t sure that I would have the commitment to do it at home, you know, life gets in the way” (ID4 Female 70-74 years FaME) (Maula et al., 2019)* | U |
|  | *“I would have rather have gone to the classes because I think it encourages you more, when you are at home you have got no stimulation” (ID30 Female 75-79 years OTAGO) (Maula et al., 2019)* | U |
|  | *“Well because you have got to be regimented yourself to do these things haven’t you? You have got to make sure you do them, my husband was alive then so I had a lot to do looking after him you see so it was a bit difficult at times yes” (ID29 Female 70-74 years OTAGO) (Maula et al., 2019)* | U |
|  | *“When there was no record at all required then I just didn’t bother doing it” (ID1 Female 70-74 years OTAGO) (Maula et al., 2019)* | U |
|  | *“And it is the company as well when you’re on your own, you have not got the company to encourage you, you know I tended to start and do the exercises but after about half an hour, I got a little bit fed up with it so I left it off and then perhaps went back to it the next day. Whereas if you’re in a class you do it in one go don’t you?” (ID30 Female 75–79 OTAGO) (Maula et al., 2019)* | U |
|  | *“My main difficulty is that life gets in the way because we have quite a busy life” (ID4 Female 70-74 years FAME) (Maula et al., 2019)* | U |
|  | *“I am a busy person, I do try and fit in as much as I can and as I say I paint and it annoys me when I haven’t got enough time to do my painting so you know I like to put some time aside to paint” (ID8 Female 75-79 years FAME) (Maula et al., 2019)* | U |
|  | *“I just think I am not that sort of person, I have got enough to do without being you know… taking exercise up. In a while I will have, I have got all of my planting to do, all my pots and things so I don’t think anything would encourage me to go to the gym or anything” (ID28 Female 75-79 years OTAGO) (Maula et al., 2019)* | U |
|  | *“would have looked to join a class with people, there is a class that is not too far from where I live and I have looked at that once or twice and thought about going but circumstances are that I just haven’t got the time.” (ID1 Female 70-74 years OTAGO) (Maula et al., 2019)* | U |
|  | *“It is still very much about understanding the aim and meaning of PA. Many persons think that a short walk with their dog is enough. But trying to make them realize that they need to increase and be active at another intensity is a challenge.” (Ehn et al., 2019)* | U |
|  | *“For the seniors we meet at the geriatric clinic, the main benefit might be just to be active during the day. Because an enormous amount of time is spent on nothing, people are very passive.” (Ehn et al., 2019)* | U |
|  | *“I guess many seniors think that they are worth sitting down, because they have worked and labored all their lives. And this I hear very often from older persons.” (Ehn et al., 2019)* | U |
|  | *“I was sitting down reading my paper and then this alarm told me I had to move. I was only trying to relax. So, I silenced the alarm and continued to read the paper” (10B) (Abouzahra and Ghasemaghaei 2020)* | U |

*Abbreviations*: U: Unequivocal findings (clearly supported by data); C: credible findings (open to interpretation); Un: Unsupported findings (lacking data support)
